# Supplementary material for: Assessing the clinical relevance of point-of-care ultrasound for hospitalists: Influence on clinical reasoning and decision-making
Source: PLoS One. 2025 Dec 18;20(12):e0338202. doi: 10.1371/journal.pone.0338202 (PMC12714290; doi:10.1371/journal.pone.0338202)
Supplement: S1 Appendix — Questionnaire completed by participants before the study. It collects demographic data, academic background, previous experience with point-of-care ultrasound (POCUS), and self-assessed confidence in performing and interpreting POCUS examinations. (DOCX) [file pone.0338202.s001.docx]

**S1 Appendix A****. Form on participants’ personal data**

| 1. **Age (in years)** |
| --- |
| 1. **Sex**  - Female - Male |
| 1. **What is your country of origin?** |
| 1. **Have you completed any undergraduate degree other than Medicine?**  - Yes - No |
| 1. **If you answered "Yes" to the previous question, please specify the completed degree** |
| 1. **Have you completed any medical residency program other than Internal Medicine?**  - Yes - No |
| 1. **If you answered "Yes" to the previous question, please specify the completed residency program.** |
| 1. **How many years ago did you complete your Internal Medicine training?** |
| 1. **Have you received prior formal theoretical and/or practical training in POCUS? Formal training includes a dedicated POCUS internship during medical school and/or residency, or a certified theoretical and/or hands-on course.**  - Yes - No |
| 1. **How many times have you used POCUS for diagnostic purposes?** |
| 1. **How confident are you in performing POCUS and interpreting the images obtained?**   **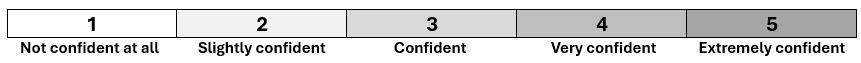**   - 1: Not confident at all - 2: Slightly confident - 3: Confident - 4: Very confident - 5: Extremely confident |
