# Supplementary material for: Assessing the clinical relevance of point-of-care ultrasound for hospitalists: Influence on clinical reasoning and decision-making
Source: PLoS One. 2025 Dec 18;20(12):e0338202. doi: 10.1371/journal.pone.0338202 (PMC12714290; doi:10.1371/journal.pone.0338202)
Supplement: S2 Appendix — Structured form used by participants after each POCUS application during clinical practice. It documents the clinical question, diagnostic hypothesis, confidence levels before and after POCUS, selected ultrasound modalities, image quality, findings, and subsequent changes in diagnosis or management. (DOCX) [file pone.0338202.s002.docx]

**S2 Appendix B. Form on POCUS clinical use**

| 1. **What is the clinical question that prompted you to use POCUS? Include objective data such as signs, symptoms, and complementary tests (if applicable).** |
| --- |
| 1. **BEFORE performing POCUS, what was your primary diagnostic hypothesis?** |
| 1. **What was your level of confidence in your primary diagnostic hypothesis BEFORE using POCUS?**   **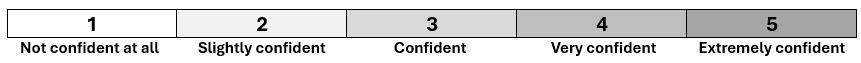**   - 1: Not confident at all - 2: Slightly confident - 3: Confident - 4: Very confident - 5: Extremely confident |
| 1. **What were the differential diagnoses BEFORE performing POCUS (if any)?** |
| 1. **What was the planned management BEFORE performing POCUS?** |
| 1. **Select the POCUS modality/modalities you performed:**  - Cardiac - Pulmonary - Abdominal - Inferior Vena Cava - Femoral and Popliteal Veins - Bladder - Other |
| 1. **If you selected "Other" above, specify the POCUS modality used.** |
| 1. **How much time (in minutes) did you spend performing POCUS?** |
| 1. **How would you describe the quality of the images you obtained?**   **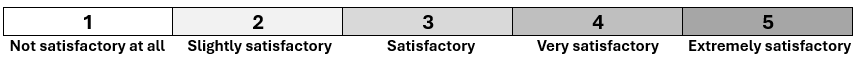**   - 1: Not satisfactory at all - 2: Slightly satisfactory - 3: Satisfactory - 4: Very satisfactory - 5: Extremely satisfactory |
| 1. **Describe the findings from the POCUS images you obtained.** |
| 1. **AFTER performing POCUS, what is your primary diagnostic hypothesis?** |
| 1. **What is your level of confidence in your primary diagnostic hypothesis AFTER using POCUS?**   **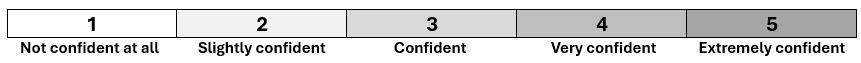**   - 1: Not confident at all - 2: Slightly confidente - 3: Confident - 4: Very confidente - 5: Extremely confident |
| 1. **Write the differential diagnoses AFTER performing POCUS (if any).** |
| 1. **What management did you implement AFTER performing POCUS?** |
