# Supplementary material for: Assessing the clinical relevance of point-of-care ultrasound for hospitalists: Influence on clinical reasoning and decision-making
Source: PLoS One. 2025 Dec 18;20(12):e0338202. doi: 10.1371/journal.pone.0338202 (PMC12714290; doi:10.1371/journal.pone.0338202)
Supplement: S3 Appendix — Survey assessing participants’ satisfaction with the POCUS course, perceived usefulness in clinical practice, importance of formal training, confidence levels after training, future interest in using POCUS, and perceived barriers to its implementation in the clinical setting. (DOCX) [file pone.0338202.s003.docx]

**S3 Appendix** **C. Form on participants' impressions and satisfaction survey**

| 1. **Rate your satisfaction with the introductory POCUS session you attended:**   **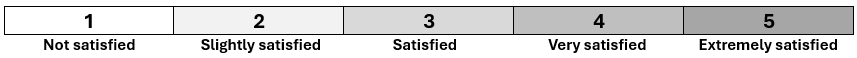**   - 1: Not satisfied - 2: Slightly satisfied - 3: Satisfied - 4: Very satisfied - 5: Extremely satisfied |
| --- |
| 1. **Indicate your agreement with the following statement: "The introductory POCUS session improved my ability to acquire and interpret ultrasound images”**   **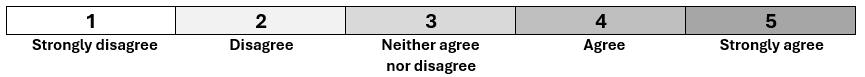**   - 1: Strongly disagree - 2: Disagree - 3: Neither agree nor disagree - 4: Agree - 5: Strongly agree |
| 1. **How useful do you consider POCUS for clinical practice on the internal medicine ward?**   **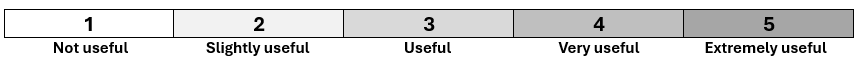**   - 1: Not useful - 2: Slightly useful - 3: Useful - 4: Very useful - 5: Extremely useful |
| 1. **In your opinion, how important is formal POCUS training in Internal Medicine residency programs?**   **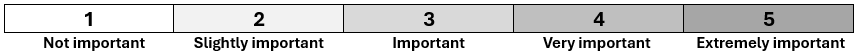**   - 1: Not important - 2: Slightly important - 3: Important - 4: Very important - 5: Extremely important |
| 1. **After the introductory POCUS session, how confident are you in operating the device and interpreting the images obtained?**   **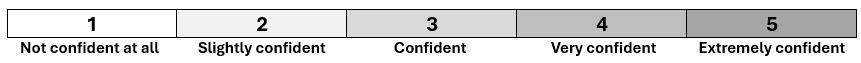**   - 1: Not confident at all - 2: Slightly confidente - 3: Confident - 4: Very confidente - 5: Extremely confident |
| 1. **How interested are you in incorporating POCUS into your future clinical practice? 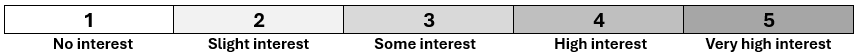**  - 1: No interest - 2: Slight interest - 3: Some interest - 4: High interest - 5: Very high interest |
| 1. **During the study period, what were your main difficulties using POCUS in clinical practice? (Structural, logistical, personal, or others)** |
