# Supplementary material for: Assessing the clinical relevance of point-of-care ultrasound for hospitalists: Influence on clinical reasoning and decision-making
Source: PLoS One. 2025 Dec 18;20(12):e0338202. doi: 10.1371/journal.pone.0338202 (PMC12714290; doi:10.1371/journal.pone.0338202)
Supplement: Attachment — Submitted filename: Response to Reviewers.docx [file pone.0338202.s004.docx]

Maíra Dias Souza
Avenida Doutor Arnaldo 455 Cerqueira Cesar - São Paulo - SP, Brazil mairadiassouza@gmail.com
30 June 2025
Editor-in-Chief PLOS ONE

Dear Editor,

We would like to express our sincere thanks to you and the reviewers for the thoughtful and constructive feedback on our manuscript. We have carefully revised the text in response to the comments and suggestions provided.

Please find below our detailed responses to the reviewers’ comments. We hope that the revised version of the manuscript now meets the expectations of the Journal.

Thank you again for your time and consideration.

Sincerely,

Maíra Dias Souza
Instituto Perdizes, Hospital das Clínicas da Faculdade de Medicina da Universidade de São Paulo, Faculdade de Medicina, Universidade de São Paulo, São Paulo, São Paulo, Brazil
mairadiassouza@gmail.com

**Response to Reviewer #1**

We would like to sincerely thank Reviewer #1 for their thoughtful and constructive comments, which have greatly improved the clarity, rigor, and overall quality of our manuscript. We carefully considered all the suggestions and revised the text accordingly. Below, we provide detailed responses to each comment.

**Study Design**

Comment: While described as an "exploratory prospective study," a more precise classification such as a "descriptive prospective study" would accurately reflect the study's aim to observe and describe the influence of POCUS.

Response: We thank the reviewer for this suggestion. We fully agree that "descriptive prospective study" more accurately reflects the design and objectives of our work. Accordingly, we revised the Methods section (line 110), replacing the previous phrasing with: “This is a descriptive prospective study”. We also made the same adjustment in the Abstract (line 33) to ensure consistency throughout the manuscript.

**Sample Size Calculation**

Comment: Clarification is needed regarding the odds ratio of 4 used in the sample size calculation. Please specify which "form" is being discussed and provide a detailed explanation of the rationale behind all values and assumptions considered for known or unknown variables in the calculation.

Response: We sincerely thank the reviewer for the careful attention dedicated to our sample size calculation, and for this valuable observation. The text we provided in the first manuscript was inaccurate. As we first designed our research project and contacted a statistician, they suggested an approach based on the McNemar test. After further discussion among the investigators, we decided to pursuit a different approach. Unfortunately, the first version of the manuscript sent to your consideration mistakenly presented the original statistical approach.

The investigators defined along with a consultant statistician a more suitable sample size approach: the formula for estimating proportions in binary nominal outcomes, with finite population correction. This method better reflects our primary outcome, defined a priori as the occurrence of a change in the main diagnostic hypothesis and/or management plan.

The updated file of our manuscript presents the rationale and the assumptions in the Methods section, between lines 165 and 174.

**Statistical Analysis**

Comment: It would be beneficial to explicitly state for which specific variables the Likert scale responses were used and subsequently grouped for analysis.

Response: We thank the reviewer for this suggestion. We agree that these variables were not clearly expressed in this section of the manuscript, and with the importance of its specification. We revised the Methods section to clearly indicate which variables were measured using Likert scales and how these responses were grouped for analysis. The following sentence was added (lines 179–184): The following variables were recoded according to this categorization approach: confidence in performing POCUS (S1 appendix A); diagnostic confidence before and after POCUS and image quality (S2 appendix B); as well as satisfaction, perceived usefulness, and importance of POCUS training; confidence in equipment use and image interpretation; and interest in future use of POCUS (S3 appendix C).

**Impact of Brief Training**

Comment: The study's premise that a single two-hour POCUS training session can instill confidence for accurate diagnosis, especially without prior formal exposure, warrants further discussion. The results indicate that 26% of participants had previous ultrasound training (median of 15 POCUS examinations prior to the study), with 68% already reporting confidence in using POCUS. This suggests a potential confounding effect from these pre-trained individuals. It would be valuable to analyze the impact of this short training separately for individuals with no prior ultrasound experience to better isolate its true effect.

Response: We thank the reviewer for this insightful observation. Indeed, we agree this hypothesis is reasonable and warrants further statistical analysis. This prompted us to further explore the potential confounding effect of prior POCUS experience. Following this suggestion, we conducted an additional analysis comparing OSVE performance between participants with and without prior formal POCUS training.

The “Statistical analysis” section was updated to include this new step (lines 196-197): “Independent samples t-tests were conducted to compare participants' pre-test and post-test OSVE performance according to prior formal POCUS training.”

The results of this analysis were incorporated into the revised manuscript (lines 225–234): “To assess the relationship between participants’ performance on the pre- and post-test OSVE and prior POCUS training, participants were divided into two groups: those with prior POCUS training and those without. No difference was observed when comparing pre-test scores (T = 0.643; p = 0.535), post-test scores (T = -0.0570; p = 0.956), and the change between pre and post-test scores (T = 0.522; p = 0.616) among both groups. To evaluate whether prior hands-on POCUS experience—measured by the number of times participants reported having performed POCUS before the study—was associated with the change in OSVE scores, we applied the Spearman rank-order correlation. No significant relationship was found between the number of prior POCUS procedures and score variation (R < 0.001; p = 1.000).”

These findings were also addressed in the Discussion (lines 476–485): “This study found no statistically significant differences in OSVE performance between participants with and without prior formal POCUS training, either in the pre-test, post-test, or in the variation between them. Likewise, no correlation was observed between the number of prior POCUS procedures performed and changes in test scores. These findings suggest that previous exposure to POCUS—whether formal or practical—was not associated with better performance in this structured assessment. A possible explanation is that the OSVE may evaluate broader skills such as clinical reasoning and integration of ultrasound findings into decision-making, which may not be fully developed through prior training alone. Additionally, variability in the type and depth of previous training, as well as the small sample size, may have limited the detection of differences.”

**Learning Curve and Supervision**

Comment: The authors' own finding that "No significant difference was found between pre-test and post-test performance (T = 105.00; p = 0.970), indicating that our single short-duration POCUS training did not improve participants' performance in image interpretation" reinforces the understanding that POCUS, despite its short learning period, typically requires supervision and multiple hands-on exposures for proficiency in image interpretation. We did not find any mention of how the POCUS findings were verified.

Response: We thank the reviewer for this important observation. We realize that our original wording may have caused ambiguity regarding what was meant by "participants' performance in image interpretation." The phrase refers specifically to participants’ performance in interpreting ultrasound images presented in the Objective Structured Video Exam (OSVE), not the interpretation of POCUS images they acquired during the study. To clarify this, we revised the sentence in the Results section (lines 463–465) as follows: “No significant difference was found between pre-test and post-test performance (T = 105.00; p = 0.970), indicating that our single short-duration POCUS training did not improve participants' performance in image interpretation on the OSVE.”

We appreciate the reviewer’s highlighting of these points, which helped improve clarity and transparency in our manuscript.

**Pre- and Post-Training Test Participation**

Comment: The limited participation rates for the pre- and post-training objective structured video examinations (OSVE) (63% and 53% respectively) could potentially dilute the observed impact of the training on participants' knowledge. This limitation should be acknowledged when discussing the effectiveness of the training.

Response: We thank the reviewer for this observation. We agree that the limited participation rates in the pre- and post-training OSVE assessments may have influenced the measured impact of the training intervention. To address this, we have explicitly acknowledged this limitation in the Discussion section (lines 471–473), adding the following statement: “Additionally, the limited participation rates in the pre- and post-training OSVE—63% and 53% respectively—may have diluted the observed impact of the training on participants' knowledge.”

This acknowledgment emphasizes the potential influence of incomplete data on the evaluation of the training’s effectiveness.

**Verification of Diagnostic and Management Changes**

Comment: The methodology for verifying the accuracy of diagnosis and management decision changes needs to be clearly explained. How were these changes objectively assessed and confirmed?

Response: We thank the reviewer for highlighting this important point. We agree that the manuscript did not clearly specify the verification process for the accuracy of diagnostic findings and management decisions influenced by POCUS. To address this, we have added a clarifying statement in the Methods section (lines 151–153), as follows: “Since POCUS images were not recorded, it was not possible to independently verify the accuracy of the diagnostic findings or the clinical management decisions informed by them.”

This addition acknowledges the study limitation regarding objective verification and improves transparency about the methodology used.

**Timing of Feedback**

Comment: The timing of feedback regarding the usefulness of POCUS in clinical practice is crucial. If the feedback was collected immediately post-training, it may not accurately reflect the sustained utility or impact. Please clarify when this feedback was gathered.

Response: We thank the reviewer for pointing out this important issue. Upon review, we recognized that the original manuscript did not clearly specify the timing of the participants’ impressions and satisfaction survey. To clarify, we removed the paragraph stating that the survey was completed immediately post-training. We replaced it with a more accurate description in lines 160–163: “Upon study completion, participants completed a survey (Fig 1) assessing their satisfaction with POCUS training, its usefulness and relevance in clinical practice, confidence in using the method, and any challenges encountered with the equipment (S3 appendix C).”

This revision reflects that feedback was collected at the end of the study, thus capturing a more comprehensive perspective on the sustained utility and impact of POCUS in clinical practice. Accordingly, we updated Figure 1 to represent this change. Additionally, due to the change in appendix citations, we reversed the order of appendices B and C to maintain consistency in the text.

We believe these clarifications enhance transparency and address the reviewer’s concern about the timing of feedback collection.

**Self-Confidence Evaluation**

Comment: It appears that self-confidence while using POCUS in clinical scenarios was evaluated in only 10 out of 19 respondents (50% of the study subjects). This smaller sample size for confidence evaluation should be highlighted as a limitation.

Response: We appreciate the reviewer’s comment regarding the sample size for the self-confidence evaluation. We agree that assessing self-confidence in only 10 out of 19 participants (53%) represents a limitation that restricts the generalizability and strength of any conclusions drawn from this analysis. Accordingly, we have explicitly acknowledged this limitation in the Discussion section (lines 496–499):

“Although no significant correlations were found between prior formal training and self-confidence, it is important to highlight that self-confidence in using POCUS in clinical scenarios was assessed in only 10 out of 19 respondents (53%), which limits the strength of conclusions regarding confidence and should be considered a limitation.”

This addition ensures transparency regarding the interpretation of our findings related to participants’ self-confidence using POCUS in clinical practice.

**Results Presentation**

**Redundancy in Reporting**

Comment: Avoid repeating numerical values in the text if they are already clearly presented in tables or figures (e.g., Figure 2, 3, 4, Table 2).

Response: We thank the reviewer for this important suggestion regarding the clarity and conciseness of our results presentation. In response, we have revised the manuscript to reduce redundancy between the text and the tables/figures, as follows:

- For **Table 1**, we removed detailed numeric descriptions from the text and instead provided a concise summary statement (lines 202–203):
  “Participant demographics and characteristics are summarized in Table 1.”
- Similarly, for **Table 2**, we streamlined the description (lines 339–341):
  “To assess participants’ performance over the course of the study, we compared the first and last POCUS clinical impact forms completed by each participant. Final forms were completed by 73.7% of them. Summary data are presented in Table 2.”
- For **Figures 2 and 3**, we opted to avoid repeating detailed data in the text and included a general reference to these figures (lines 254–255):
  “Among the 104 forms, POCUS was used by participants in various modalities and for multiple clinical purposes, as summarized in Figs 2 and 3, respectively.”
- However, for **Figures 1, 4, and 5**, we chose to maintain both textual descriptions and the figures themselves. We believe this approach enhances the reader’s understanding of the study methods and results, as the textual explanations complement the visual data presentation.

These revisions improve the manuscript’s readability by eliminating unnecessary repetition while preserving clarity and comprehension.

**POCUS Utilization Data**

Comment: While the presentation of organ evaluations (lines 237–246) provides some detail, a more impactful representation of POCUS clinical uses could be achieved by focusing on the 167 items for clinical uses depicted in lines 251–258, as these directly address the clinical questions POCUS is designed to answer.

Response: We appreciate the reviewer’s insightful suggestion to emphasize the clinical uses of POCUS over organ-specific evaluations, as this better reflects the practical utility of the method in answering bedside clinical questions. In accordance with this recommendation, we have revised the discussion section by removing the paragraph originally between lines 501 and 506, which described the frequency of organ evaluations. This paragraph was replaced by a more concise statement (lines 451–453):

“POCUS was applied across diverse clinical contexts, including cardiopulmonary, urinary, and hemodynamic assessments, underscoring its versatility in hospital-based practice.”

This change allows us to focus the discussion on the broad clinical applicability of POCUS, highlighting its role in guiding patient care decisions rather than solely detailing the anatomical areas assessed. We believe this adjustment better captures the essence of POCUS utilization and aligns with the reviewer’s recommendation.

**Time Efficiency Analysis**

Comment: In the "Time spent per number of modalities" assessment, the results section only states "The total time spent using POCUS was 935 minutes, with a mean value of 9 minutes per patient and 5.3 minutes per modality performed." It would be valuable to evaluate the effect of prior POCUS exposure on time efficiency.

Response: We appreciate the reviewer’s suggestion to further explore the influence of prior POCUS exposure on time efficiency. Accordingly, we conducted an additional analysis, which has been incorporated into the Results section (lines 264–273). Specifically, we assessed the total time spent per modality in relation to participants’ previous POCUS experience, measured both by self-reported number of exams performed before the study and formal training status.

Our findings indicate that participants with prior formal POCUS training (n = 5) had a median time per modality of 8.2 minutes, compared to 6.7 minutes among those without formal training (n = 14). However, this difference was not statistically significant (Mann-Whitney Rank Sum test, T = 49.000; p = 0.921). Notably, Spearman rank correlation analysis demonstrated a significant negative correlation between the number of prior POCUS exams performed and time spent per modality (R = -0.559; p = 0.00127), suggesting that greater hands-on experience was associated with increased time efficiency.

These results provide valuable insights into how practical exposure to POCUS can enhance procedural speed, even if formal training alone did not significantly affect the time spent. We have included this discussion in the manuscript to better contextualize the factors influencing time efficiency in POCUS use.

**Elaboration on Diagnostic Hypothesis Changes**

Comment: In Results Line 330, the statement "In all cases where the primary diagnostic hypothesis changed, the POCUS findings were inconsistent with what was expected" requires further explanation for clarity.

Response: We thank the reviewer for highlighting the need for greater clarity in this statement. Our intended meaning was that in every case where participants altered their primary diagnostic hypothesis after performing POCUS, the ultrasound findings did not match the participants’ initial diagnostic expectations. To make this clearer, we have revised the sentence in the Results section (lines 335–337) to:
"In all cases where the primary diagnostic hypothesis changed, the POCUS findings differed from the participants’ initial expectations."
This wording more precisely reflects that the change in diagnostic hypothesis was triggered by unexpected ultrasound findings that challenged the participants’ original assumptions.

**Table 2**
Comment: Consider whether Table 2 is truly necessary, as the data it presents does not appear to reflect significant findings that warrant a separate table.

Response: We appreciate the reviewer’s suggestion regarding Table 2. After careful consideration, we decided to retain this table in the manuscript. Since we have removed much of the descriptive text about these results to avoid redundancy, the table becomes essential for presenting the comparison between the first and last clinical impact forms clearly and concisely. Moreover, we believe that displaying these findings in a table format facilitates readers’ understanding by allowing an immediate visual comparison of the data, which might be more cumbersome to convey solely through narrative text. Therefore, Table 2 remains a valuable component for effectively communicating these specific study results.

**Figures and Tables Placement**

Comment: For ease of review, tables and figures should be provided separately from the main text, rather than embedded within it.

Response: We appreciate the reviewer’s suggestion to adapt the file so it could be easier for reviewers to evaluate our text, tables and figures. Unfortunately, we did not make all these changes.

Regarding tables, we have followed the specific instructions previously received from PLOS ONE editorial staff, who requested that tables be included within the main manuscript text during submission. Therefore, we have embedded all tables within the text accordingly. On the other hand, submitted separately. This decision was made in compliance with the Journal guidelines for authors. We hope this arrangement meets the journal’s standards for clarity and ease of review.

**Discussion and Conclusion
Systematic use of POCUS**

Comment: The discussion could benefit from emphasizing the importance of systematic POCUS use to enable standardized application of this widely available tool. Referencing relevant literature on systematic POCUS approaches in various subspecialties (e.g., Pokharel B. Systematic use of Point of Care Ultrasound in Neurosurgical Intensive Care Unit: a practical approach. Quant Imaging Med Surg 2023;13(4):2287-2298. <https://dx.doi.org/10.21037/qims-22-667>) could strengthen this point.

Response: We agree with the reviewer on the importance of highlighting the role of systematic POCUS use in improving clinical practice. Accordingly, we have incorporated this perspective into the discussion (lines 453–456), stating: "While our study did not evaluate the impact of a systematic approach, the breadth of its use observed is consistent with existing literature that supports structured protocols to improve the consistency and diagnostic accuracy of POCUS [131–136]. Such approaches may enhance bedside clinical decision-making, particularly in acute care settings."

We also included recent references to underscore how standardized POCUS protocols across various specialties have been shown to improve clinical outcomes, thereby reinforcing the relevance of adopting systematic approaches in hospital-based care.

**Incidental Diagnoses**

Comment: The finding of incidental diagnoses in 8% of cases is interesting, but it also raises a valid concern. POCUS is primarily designed to answer specific clinical questions in a binary format, not for comprehensive organ or system evaluations. This finding could be discussed in the context of the potential for POCUS use to become distracted from its primary purpose.

Response: We appreciate the reviewer’s insightful observation regarding incidental findings. We have addressed this point in the discussion section (lines 422–428) as follows: "However, the identification of incidental findings also raises an important consideration. Such findings may divert the use of POCUS from its primary purpose—providing rapid answers to focused, binary clinical questions at the bedside—toward a more exploratory or comprehensive approach that it is not designed to fulfill. This highlights the need to maintain a clear diagnostic focus and to apply POCUS judiciously, ensuring it enhances rather than detracts from clinical reasoning."

This addition emphasizes the importance of balancing the diagnostic utility of POCUS with the risk of overextending its scope beyond its intended clinical role.

**Learning Curve for Less Experienced Individuals**

Comment: In the Discussion, line 474, the deduction that "participants showed a trend toward increased efficiency in POCUS execution over time, particularly those with less prior exposure, suggesting a learning curve" is an important point. However, to fully support this claim, the authors should separate the analysis of efficiency for groups with and without prior POCUS experience.

Response: We thank the reviewer for this valuable suggestion. In response, we performed a separate analysis to assess participants’ efficiency over time according to prior POCUS experience and formal training. This analysis is now described in the Results section (lines 359-371): "Participants' performance over time was analyzed relative to their prior experience and formal POCUS training. Prior experience was measured by the self-reported number of times each participant had performed POCUS before the study. Performance over time was assessed by calculating the variation in the ratio of time spent per modality between the first and last forms completed by each participant. The Mann-Whitney Rank Sum test was used to compare changes in time efficiency between participants with and without prior formal POCUS training, revealing no statistically significant difference (T = 55.000; p = 0.674). Spearman’s rank-order correlation revealed a significant negative correlation between prior POCUS experience and the variation in time-per-modality ratio between the first and last forms (R = -0.570; p = 0.011). Additionally, significant negative correlations were found between prior experience and the time-per-modality ratio in both the first form (R = -0.548; p = 0.015) and the last form (R = -0.585; p = 0.035)."

To avoid redundancy, we removed the previously included paragraph reporting similar correlations.

In the Discussion (lines 487–495), we expanded on these findings: "While prior formal POCUS training was not associated with improvements in time efficiency—measured by the change in time-per-modality ratio between each participant’s first and last form—greater hands-on experience, defined as the self-reported number of times participants had performed POCUS prior to the study, showed a significant negative correlation with this variable. This means that participants with more prior POCUS experience not only started out faster (lower time-per-modality ratios in their first forms) but also showed greater improvement in efficiency over time. These findings suggest that repeated practical exposure may play a more important role in enhancing efficiency during ultrasound use than formal training alone”

We believe this additional analysis strengthens our conclusions regarding the learning curve and efficiency in POCUS performance.

**Conclusion**

Comment: The conclusion should primarily summarize the study's key findings based on the results, and limitations should not be reiterated in this section.

Response: We thank the reviewer for this important observation. Accordingly, we have revised the concluding paragraph to focus on future directions without reiterating limitations. Specifically, we edited the paragraph (lines 535–538) to read: "Nevertheless, future research should address whether enhancements in clinical reasoning facilitated by POCUS translate into measurable improvements in patient outcomes and healthcare efficiency, such as reduced hospital length of stay, readmission rates, or mortality."

This revision removes restatement of study limitations from the conclusion, thereby emphasizing the study’s implications and areas for further investigation.

**Response to Reviewer #2**

We would like to sincerely thank Reviewer #2 for the thoughtful and positive evaluation of our manuscript. We are very pleased that the reviewer found our study to be well-structured and relevant, and that it contributes to a better understanding of the role of POCUS in clinical settings with limited resources.

We also appreciate the insightful suggestions for future research directions, which we consider valuable and plan to explore in future studies.
