# Supplementary material for: Assessing the clinical relevance of point-of-care ultrasound for hospitalists: Influence on clinical reasoning and decision-making
Source: PLoS One. 2025 Dec 18;20(12):e0338202. doi: 10.1371/journal.pone.0338202 (PMC12714290; doi:10.1371/journal.pone.0338202)
Supplement: Attachment — Submitted filename: PlosOne_Report.docx [file pone.0338202.s005.docx]

This manuscript addresses an important and timely question regarding the impact of point-of-care ultrasound (POCUS) training on clinical decision-making in transitional care units with limited imaging resources. The focus on hospitalists’ diagnostic reasoning, management decisions, and confidence levels is relevant to both clinical practice and medical education.

The chosen study design, however, requires clarification and more precise description. The manuscript currently refers to the study in broad terms, but given the structure — participants undergoing baseline assessment, receiving an educational intervention (POCUS training), and then being reassessed with both OSVE and questionnaires — this aligns most accurately with a prospective quasi-experimental before-and-after design.

This classification is important because the study does involve an intervention and measures outcomes before and after its implementation, but it does not include randomization or a parallel control group. Calling it a ‘descriptive prospective study’ would be misleading, as descriptive studies do not assess causal effects or intervention impact. Properly labeling the design as quasi-experimental strengthens methodological transparency and allows readers to appropriately interpret the results.

I have concerns regarding the measurement of two key process variables: (a) time spent performing the POCUS exam, and (b) quality of images obtained. According to the data collection form, both variables were captured through participant self-report rather than objective measurement. For time spent, it is unclear whether this was based on a stopwatch or structured observation; as presented, the data seem to rely entirely on participants’ subjective estimation. Similarly, the assessment of image quality was based on participants’ own descriptions without saving or independently reviewing the images. This introduces a high risk of recall bias and self-report bias. Moreover, not storing images for subsequent blinded quality review represents a missed opportunity to validate this outcome objectively. These limitations should be explicitly acknowledged in the manuscript, as they constrain the strength of the conclusions regarding feasibility and quality of POCUS implementation.

In addition, the manuscript does not provide sufficient information on the development and validation of the data collection forms. It is unclear whether the instruments underwent a systematic process of construction (e.g., based on literature review, expert consultation) and whether any procedures to establish evidence of content validity were performed. Without such information, it is difficult to assess whether the forms adequately captured the intended constructs (e.g., diagnostic hypotheses, management changes, confidence levels, time spent, and image quality). The absence of reported validity evidence represents an important limitation, as the accuracy and interpretability of the findings rely heavily on the adequacy of these instruments.

In addition, it is unclear whether the POCUS training and subsequent examinations followed a standardized protocol, such as the RUSH protocol. The data collection form included the following examination categories: cardiac, pulmonary, abdominal, inferior vena cava, femoral and popliteal veins, bladder, and ‘other’. The RUSH protocol encompasses cardiac function and contractility assessment, evaluation of intravascular volume status via the inferior vena cava, identification of free fluid in the abdomen, lung assessment for pneumothorax or pulmonary embolism, and lower limb DVT screening. Given this overlap, it should be explicitly stated whether the investigators adopted the RUSH protocol in its entirety, used a modified version, or employed another structured approach. If the RUSH protocol was not used, the authors should clearly describe which anatomical regions and clinical targets were prioritized in both training and clinical application.

The manuscript states that responses to open-ended questions were kept in their original format, but it does not describe the procedures adopted for their analysis. It remains unclear whether these responses were analyzed qualitatively (e.g., through thematic or content analysis), coded systematically by independent reviewers, or simply presented as anecdotal remarks. Without a clear description of the analytic approach, the contribution of these data to the study’s findings is difficult to assess. The authors should specify the method used to analyze the open-ended responses, including whether any coding framework, inter-rater reliability, or software tools were applied. If the responses were not systematically analyzed, this should be acknowledged as a limitation.

The sample size rationale estimates a single post-POCUS proportion with 95% confidence and 10% margin of error, assuming p=0.20, yielding ≈62 forms. This is acceptable for estimating a single proportion. However, because multiple forms were submitted by the same hospitalists, observations are clustered. The calculation should therefore include a design-effect inflation based on the average forms per clinician and an assumed ICC. Using 5.5 forms/physician and plausible ICCs (0.05–0.10) increases the requirement to approximately 76–90 forms. The 104 forms collected likely satisfy this, but the manuscript should explicitly report the clustering adjustment (DEFF and ICC). In addition, the finite-population correction used should reflect the finite number of eligible encounters in the unit rather than the number of internists in the state; otherwise, FPC should be omitted.

The study demonstrates positive trends but does so with methodological fragility. The results should be framed more cautiously, emphasizing feasibility and potential utility rather than efficacy. Stronger study designs (e.g., randomized controlled trials or controlled before-and-after studies with validated outcome measures and objective performance data) would be necessary to substantiate these findings.
