# Supplementary material for: Assessing the clinical relevance of point-of-care ultrasound for hospitalists: Influence on clinical reasoning and decision-making
Source: PLoS One. 2025 Dec 18;20(12):e0338202. doi: 10.1371/journal.pone.0338202 (PMC12714290; doi:10.1371/journal.pone.0338202)
Supplement: Attachment — Submitted filename: Response_to_Reviewers_auresp_2.docx [file pone.0338202.s006.docx]

Maíra Dias Souza
Avenida Doutor Arnaldo 455 Cerqueira Cesar - São Paulo - SP, Brazil mairadiassouza@gmail.com
23 September 2025
Editor-in-Chief PLOS ONE

Dear Editor,

We would like to express our sincere thanks to you and the reviewers for the thoughtful and constructive feedback on our manuscript. We have carefully revised the text in response to the comments and suggestions provided.

Please find below our detailed responses to the reviewers’ comments. We hope that the revised version of the manuscript now meets the expectations of the Journal.

Thank you again for your time and consideration.

Sincerely,

Maíra Dias Souza
Instituto Perdizes, Hospital das Clínicas da Faculdade de Medicina da Universidade de São Paulo, Faculdade de Medicina, Universidade de São Paulo, São Paulo, São Paulo, Brazil
mairadiassouza@gmail.com

**Response to Reviewer #3**

We thank the reviewer for the insightful comments and for highlighting essential patient-safety considerations related to POCUS training and verification. We revised the manuscript to clarify the scope of our educational intervention and the interpretability of our findings.

**Comment 1:**
*“POCUS education for 120 minutes without a follow up period of verification of competency or progression of skill.”*

We agree that a brief session does not establish technical competency. In the revised Methods, we clarify that the 120-minute session functioned as a brief orientation to align participants on basic Point-of-care Ultrassound (POCUS) concepts, terminology, and device operation, and was not designed to establish or verify technical competency. No follow-up skill verification or assessment of progression was performed, and the session was not analyzed as an intervention. Additionally, to streamline the manuscript and keep focus on the primary objectives, we removed the Objective Structured Video Examination (OSVE) and all related pre/post analyses, as well as the exploratory comparison between the first and last POCUS forms and the derived efficiency metric (“time spent per number of modalities”). These changes prevent secondary analyses of educational (OSVE) and process measures from overshadowing our primary per-encounter descriptions of how clinician-directed POCUS related to diagnostic hypotheses, management decisions, and confidence during routine care.

**Edits implemented:**

- **Abstract – Materials and Methods:**
  - **Edited:**
    - **Original:** “This descriptive prospective study enrolled 19 hospitalists **who underwent a brief POCUS training session**. **Participants completed objective structured video examinations (OSVE) before and after training**, and used POCUS in their clinical practice, documenting each application with a standardized form detailing diagnostic hypotheses, management, and confidence levels. Changes in these parameters following POCUS use in real patients were analyzed.”.
    - **Revised to:** “Prospective descriptive study in a transitional care unit enrolling 19 hospitalists. During routine care, clinicians used POCUS at their discretion and, immediately afterward, completed a structured form capturing the clinical question, pre- and post-POCUS diagnostic hypotheses, confidence levels, and management decisions. Per-encounter changes in these parameters were summarized descriptively.”.
- **Abstract - Results:**
  - **Removed:** “While a short training session did not significantly improve OSVE scores, participants reported high satisfaction with the training and perceived utility of POCUS.”.
- **Main text – Materials and Methods (Participants and Procedures):**
  - **Removed:** “The principal investigator then conducted an initial objective video assessment (OSVE) with 20 questions (maximum score of 10 points) to evaluate participants’ ultrasound interpretation skills (Fig 1).”.
  - **Edited:**
    - **Original:** “Fig 1: Study procedures flowchart. Hospitalists who agreed to participate signed an informed consent form (ICF) and completed a personal data form. **Participants underwent initial objective video assessments (OSVE) and received point-of-care ultrasound (POCUS) training. Following this, they completed a second OSVE** **and a questionnaire assessing their personal impressions and satisfaction.** Each time POCUS was utilized in clinical practice, a detailed record was made using a standardized form.”.
    - **Revised to:** “Fig 1: Study procedures flowchart**.** Hospitalists who agreed to participate signed an informed consent form (ICF) and completed a personal data form. **They then attended a brief POCUS orientation.** During routine care, POCUS was used at clinicians’ discretion. Immediately after each use, participants completed a structured encounter form documenting the clinical question, pre- and post-POCUS diagnostic hypotheses, confidence levels, and management decisions. **At the end of the study, participants completed a perceptions/satisfaction survey.**”.
  - **Edited:**
    - **Original:** “Following the initial assessment, all participants underwent **a 120-minute theoretical and practical POCUS training sessio**n (Fig 1). Topics covered included POCUS applications in clinical practice, equipment use and maintenance, transducer types, image optimization, and interpretation of images from lungs, pleura, heart, abdomen, and vessels. **After training sessions, participants completed a second OSVE to re-evaluate their ultrasound interpretation skills** (Fig 1).”.
    - **Revised to:** “Following the initial assessment, all participants underwent **an introductory educational session on POCUS** (Fig 1), which covered basic applications in clinical practice, equipment use and maintenance, transducer types, image optimization, and interpretation of images from lungs, pleura, heart, abdomen, and vessels. **This session was not based on a standardized protocol; rather, it provided a broad overview of commonly relevant organ systems to internal medicine hospitalists. The emphasis was on introducing participants to the potential clinical applications of POCUS rather than on training them to follow a fixed, structured protocol**.”.
- **Main text – Materials and Methods (Statistical analysis):**
  - **Edited:**
    - **Original:** “**The Mann-Whitney Rank Sum test was utilized to examine participants' performance on the OSVE before and after POCUS training, as well as to analyze the “time spent per number of modalities” reported in the POCUS clinical impact forms, in relation to prior hands-on experience and formal POCUS training. The Kruskal-Wallis One-Way Analysis of Variance on Ranks was applied to compare participants’ confidence in their primary diagnostic hypothesis in the first and last forms. Independent samples t-tests were conducted to compare participants' pre-test and post-test OSVE performance according to prior formal POCUS training.** Spearman rank correlation was performed to evaluate relationships between the **remaining variables**.”.
    - **Revised to:** “Spearman rank correlation was performed to **explore associations between continuous and ordinal variables**.”.
- **Main text – Results (Pre and post-tests results):**
  - **Removed:** “For the pre- and post-training test forms (maximum score: 10), 63% (12/19) completed the pre-test, with a median score of 7.25 [5.75; 8.25]. Fifty-three per cent (10/19) completed the post-test, with a median score of 6.25 [5.0; 9.0]. The Mann-Whitney Rank Sum test showed no significant difference between pre-test and post-test performance (T = 105.00; p = 0.970). No significant correlations were found between pre-test scores and participant’s age (R = -0.163; p = 0.603), time since completion of Internal Medicine residency (R = 0.106; p = 0.733), or formal POCUS training (R = -0.155; p = 0.619). No significant correlations were found between post-test scores and participant’s age (R = 0.0864; p = 0.785), time since completion of Internal Medicine residency (R = 0.0308; p = 0.919), or formal POCUS training (R = 0.107; p = 0.759). To assess the relationship between participants’ performance on the pre- and post-test OSVE and prior POCUS training, participants were divided into two groups: those with prior POCUS training and those without. No difference was observed when comparing pre-test scores (T = 0.643; p = 0.535), post-test scores (T = -0.0570; p = 0.956), and the change between pre and post-test scores (T = 0.522; p = 0.616) among both groups. To evaluate whether prior hands-on POCUS experience—measured by the number of times participants reported having performed POCUS before the study—was associated with the change in OSVE scores, we applied the Spearman rank-order correlation. No significant relationship was found between the number of prior POCUS procedures and score variation (R < 0.001; p = 1.000).”.
- **Main text – Results (Clinical applications of POCUS):**
  - **Removed:** “To explore whether prior POCUS exposure influenced time efficiency, we analyzed the total time spent per modality for each participant in relation to previous POCUS experience —defined as the self-reported number of exams performed before the study— and formal training. Participants with prior formal POCUS training (n = 5) had a median time per modality of 8.2 minutes, compared to 6.7 minutes among those without formal training (n = 14). However, according to the Mann-Whitney Rank Sum test, this difference was not statistically significant (T= 49.000; p = 0.921). A Spearman rank correlation analysis revealed a significant negative correlation between the number of previous POCUS exams performed and the time spent per modality (R = -0.559, p = 0.00127).”.
- **Main text – Results (Evolution of performance and confidence during the study):**
  - **Removed:** “To assess participants’ performance over the course of the study, we compared the first and last POCUS clinical impact forms completed by each participant. Final forms were completed by 73.7% of them. Summary data are presented in Table 2. The Kruskal-Wallis One-Way Analysis of Variance on Ranks showed no statistically significant difference in confidence levels between the first and last forms (H = 5.791; P = 0.122). The ratio between time spent per number of modalities was stablished as an efficiency parameter — with lower values indicating greater efficiency. Spearman rank correlation analysis revealed a significant negative correlation between the efficiency in the first form and the post-test score (R = -0.671; p = 0.0290). Participants' performance overtime was also analyzed in relation to their prior experience and formal POCUS training. Prior experience was measured by the self-reported number of times each participant had performed POCUS before the study. Performance over time was assessed by calculating the variation in the ratio of time spent per modality between the first and last forms completed by each participant. The Mann-Whitney Rank Sum test was used to compare changes in time efficiency between participants with and without prior formal POCUS training, and no statistically significant difference was observed between the groups (T = 55.000; p = 0.674). Spearman’s rank-order correlation revealed a significant negative correlation between prior POCUS experience and the variation in time-per-modality ratio between the first and last completed forms (R = -0.570; p = 0.011). Additionally, significant negative correlations were found between prior experience and the time-per-modality ratio in both the first form (R = -0.548; p = 0.015) and the last form (R = -0.585; p = 0.035).”.
  - **Removed: “Table 2.** **Comparison of participants’ performance and confidence between the first and last forms on POCUS clinical impact.** The table presents changes in confidence, number of POCUS modalities performed, exam duration, image quality, and confidence in the primary diagnostic hypothesis before and after POCUS, with values shown as medians and interquartile ranges (IQR).”.
- **Main text - Discussion:**
  - **Edited:**
    - **Original: “**They had limited formal POCUS training, and their practical experience varied, **leading to differences in skill and confidence**. **While some demonstrated proficiency, many struggled with competence and self-assurance.”.**
    - **Revised to: “**They had limited formal POCUS training, and their practical experience varied, **leading to differences in confidence**.**”.**
  - **Removed:** “No significant difference was found between pre-test and post-test performance (T = 105.00; p = 0.970), indicating that our single short-duration POCUS training did not improve participants' performance in image interpretation on the OSVE. This suggests that the skills required for POCUS image interpretation likely demand more extensive training and hands-on practice. Additionally, several factors may have contributed to this finding. First, our study was not specifically designed as a medical education study, which may have impacted on the effectiveness of the training approach. Second, the assessment tool we used may have been too challenging, as reflected in the participants' test scores, potentially limiting the ability to detect improvements. Additionally, the limited participation rates in the pre- and post-training OSVE —63% and 53% respectively—may have diluted the observed impact of the training on participants' knowledge. Finally, the alignment between the assessment tool and the content covered in the training may not have been optimal, which could have influenced the measured outcomes. This study found no statistically significant differences in OSVE performance between participants with and without prior formal POCUS training, either in the pre-test, post-test, or in the variation between them. Likewise, no correlation was observed between the number of prior POCUS procedures performed and changes in test scores. These findings suggest that previous exposure to POCUS—whether formal or practical—was not associated with better performance in this structured assessment. A possible explanation is that the OSVE may evaluate broader skills such as clinical reasoning and integration of ultrasound findings into decision-making, which may not be fully developed through prior training alone. Additionally, variability in the type and depth of previous training, as well as the small sample size, may have limited the detection of differences. While prior formal POCUS training was not associated with improvements in time efficiency—measured by the change in time-per-modality ratio between each participant’s first and last form—greater hands-on experience, defined as the self-reported number of times participants had performed POCUS prior to the study, showed a significant negative correlation with this variable. This means that participants with more prior POCUS experience not only started out faster (lower time-per-modality ratios in their first forms) but also showed greater improvement in efficiency over time. These findings suggest that repeated practical exposure may play a more important role in enhancing efficiency during ultrasound use than formal training alone.”.
  - **Edited:**
    - **Original:** “**Despite the lack of statistical improvement in confidence**, the survey results revealed a highly positive perception of POCUS training.”.
    - **Revised to:** “Furthermore, the survey results revealed a highly positive perception of brief POCUS orientation session.”.

**Comment 2:**
*“After completion of the education, the participant images were not reviewed for accuracy, completeness or appropriate interpretation in the clinical context.”*

We agree and have made this limitation explicit in both the Methods and the Limitations sections, and we also reiterate it in the Conclusion to ensure a cautious interpretation of the findings.

**Edits implemented:**

- **Materials and Methods (Participants and procedures):**
  - **Edited:**
    - **Original:** “Since POCUS images were not recorded, it was not possible to independently verify the accuracy of the diagnostic findings or the clinical management decisions informed by them.”.
    - **Revised to:** “In addition, because POCUS images were not recorded, independent verification of diagnostic accuracy, image quality, and related clinical decisions was not feasible.”.
- **Discussion:**
  - **Edited:**
    - **Original:** “The limitations of this study include the lack of assessment of the impact of POCUS on robust clinical outcomes, such as length of stay, readmission rates, and mortality. Another limitation is the sample size, which may affect the generalizability of the results and increase susceptibility to bias. Additionally, the POCUS images were not recorded, which precluded independent verification of image acquisition quality and the accuracy of the corresponding diagnostic findings or management decisions. Consequently, we were unable to assess interobserver reliability or confirm whether image interpretation was consistent with accepted diagnostic criteria. Finally, the absence of gold-standard complementary exams to confirm POCUS findings represents an important limitation, as interpretation can be influenced by operator experience and patient conditions.”.
    - **Revised to:** “This study has some limitations. First, we did not assess the impact of POCUS on robust clinical outcomes such as length of stay, readmission rates, or mortality. Second, the small sample size may limit the generalizability of the results and increase susceptibility to bias. **Third, POCUS images were not recorded, precluding independent review of acquisition and interpretation, and preventing evaluation of diagnostic accuracy, interobserver reliability,** **or concordance with gold-standard criteria. Decisions were therefore based solely on participants’ self-reported findings, which may have led to overestimation of perceived safety and influence.** **In addition, process variables such as exam duration and image quality were also self-reported rather than objectively measured. Exam time was estimated by participants, raising the risk of recall bias, while image quality was judged subjectively without external validation, introducing self-report bias.** Another limitation relates to the data collection form, which was developed by the authors based on previous studies and study objectives but was not formally validated; as such, the accuracy and interpretability of the captured constructs may be constrained. Finally, open-ended responses were categorized by a single investigator without independent coding or inter-rater reliability assessment, which may introduce subjective bias. **These aspects should be considered when interpreting the feasibility and quality of POCUS implementation in our study.**”.
- **Conclusion:**
  - **Edited:**
    - **Original:** “Nevertheless, future research should address whether enhancements in clinical reasoning facilitated by POCUS translate into measurable improvements in patient outcomes and healthcare efficiency, such as reduced hospital length of stay, readmission rates, or mortality.”.
    - **Revised to: “**Future research **should employ stronger designs, such as randomized controlled trials or controlled before-and-after studies with validated outcome measures and objective performance data**, to determine whether enhancements in clinical reasoning facilitated by POCUS translate into measurable improvements in patient outcomes and healthcare efficiency, such as reduced hospital length of stay, readmission rates, or mortality.”.
  - **Edited:**
    - **Original: “**Overall, the results underscore the relevance of POCUS in supporting diagnostic accuracy and decision-making in hospital medicine. As clinical demands continue to evolve, incorporating effective bedside approaches remains essential for optimizing patient care.”.
    - **Revised to: “**Overall, the results **should be interpreted as exploratory, emphasizing feasibility and potential utility rather than efficacy**. As clinical demands continue to evolve, incorporating effective bedside approaches like POCUS could ultimately play a supportive role in improving patient care.”.

**Comment 3:**
*“Due to these two issues, the current conclusion is that 120 mins of education without verification of skills is appropriate for patient management and skill application into patient care. This is extremely concerning for me because there is a likelihood of harm reaching to the patient. I am not comfortable with that conclusion and possible outcome.”*

We fully agree and have revised the **entire manuscript** to adopt a more cautious framing. The revised text emphasizes feasibility and perceived influence on clinical reasoning, rather than suggesting effectiveness of training. We now state that stronger study designs with validated protocols and competency assessment are required to confirm these findings.

**Edits implemented:**

- **Article title:**
  - **Edited:**
    - **Original:** “Assessing the clinical relevance of point-of-care ultrasound for hospitalists: **impact** on clinical reasoning and decision-making”.
    - **Revised to:** “Assessing the clinical relevance of point-of-care ultrasound for hospitalists: **influence** on clinical reasoning and decision-making”.
- **Abstract - Introduction:**
  - **Edited:**
    - **Original: “**Point-of-care ultrasonography (POCUS) is increasingly recognized in internal medicine, yet its **impact** on hospitalists' clinical reasoning remains underexplored.”
    - **Revised to: “**Point-of-care ultrasonography (POCUS) is increasingly recognized in internal medicine, yet its **influence** on hospitalists' clinical reasoning remains underexplored.”
- **Abstract - Results:**
  - **Edited:**
    - **Original:** “Notably, POCUS had no discernible **impact** in only 13% (14/104) of cases.”.
    - **Revised to:** “Notably, POCUS had no discernible **influence** in only 13% (14/104) of cases.”.
- **Abstract - Conclusion:**
  - **Edited:**
    - **Original:** “This study **demonstrates** that POCUS **significantly influences** clinical reasoning and decision-making among hospitalists in a setting with limited imaging resources.”.
    - **Revised to: “**This study **suggests** that POCUS **may influence** clinical reasoning and decision-making among hospitalists in a setting with limited imaging resources.”
  - **Edited:**
    - **Original:** “These findings **highlight** the valuable role of POCUS in enhancing bedside **diagnostics** and patient management in internal medicine, underscoring the need for its integration into training programs and clinical practice.”.
    - **Revised to:** “These findings **suggest** that POCUS **may play a supportive** role in bedside **assessment** and patient management in internal medicine, underscoring the need for further research and structured training **before broader integration**.”.
- **Main text - Introduction:**
  - **Edited:**
    - **Original: “**Although studies suggest that POCUS leads to unexpected diagnoses and management changes among hospitalists, its direct **impact** on clinical reasoning remains understudied.”.
    - **Revised to: “**Although studies suggest that POCUS leads to unexpected diagnoses and management changes among hospitalists, its direct **influence** on clinical reasoning remains understudied.”.
- **Main text subtitles - Results:**
  - **Edited:**
    - **Original: “Impact** of POCUS on clinical reasoning and decision-making”
    - **Revised to: “Influence** of POCUS on clinical reasoning and decision-making”
- **Main text – Results (Clinical application of POCUS):**
  - **Edited:**
    - **Original: “**In total, 104 forms on POCUS clinical **impact** were completed by 19 participants throughout the study…”.
    - **Revised to: “**In total, 104 forms on POCUS clinical **use** were completed by 19 participants throughout the study…”.
- **Main text – Results (Influence of POCUS on clinical reasoning and decision making):**
  - **Edited:**
    - **Original: “**Of the 104 completed forms, only 14/104 (13%) showed no **impact** of POCUS on clinical reasoning or decision-making.”.
    - **Revised to: “**Of the 104 completed forms, only 14/104 (13%) showed no **influence** of POCUS on clinical reasoning or decision-making.”.
  - **Edited:**
    - **Original:** “Fig. 4: Diagnostic **impact**”.
    - **Revised to**: “Fig. 4: Diagnostic **influence**”.
  - **Edited:**
    - **Original: “**The remaining 24/25 (96%) recorded **significant** increases…”.
    - **Revised to: “**The remaining 24/25 (96%) **recorded** increases…”.
- **Main text - Discussion:**
  - **Edited:**
    - **Original: “**Our study **demonstrates** that POCUS **has a significant impact** on clinical reasoning and decision-making.”
    - **Revised to: “**Our study **suggests** that POCUS **may** influence clinical reasoning and decision-making.”
  - **Edited:**
    - **Original: “**Compared to Cid-Serra et al. [72], who conducted a systematic review encompassing six studies with a total of 1,836 patients, our study found a higher rate of primary diagnostic hypothesis changes (37% vs. 18%), though clinical management **impact** was similar (39% vs. 37–52.1%).”
    - **Revised to:** “Compared to Cid-Serra et al. [72], who conducted a systematic review encompassing six studies with a total of 1,836 patients, our study found a higher rate of primary diagnostic hypothesis changes (37% vs. 18%), though clinical management **influence** was similar (39% vs. 37–52.1%).”
  - **Edited:**
    - **Original: “**Similarly, our results exceeded those of Andersen et al. [120], who assessed 199 patients examined by medical residents. They reported changes in the primary diagnosis in 6.5% of cases, while the overall **impact** on clinical management was comparable (35% vs. 39%).”
    - **Revised to: “**Similarly, our results exceeded those of Andersen et al. [113], who assessed 199 patients examined by medical residents. They reported changes in the primary diagnosis in 6.5% of cases, while the overall **influence** on clinical management was comparable (35% vs. 39%).”
  - **Edited:**
    - **Original: “**These findings add to the limited body of literature on the application of POCUS in internal medicine, **supporting** its utility as a bedside modality. By demonstrating **its influence** on clinical judgment and reasoning—particularly in resource-limited settings—this study **supports its broader integration into hospitalist practice and highlights the need for standardized training**.”.
    - **Revised to: “**These findings add to the limited body of literature on the application of POCUS in internal medicine, **indicating its potential value** as a bedside modality. By illustrating **its possible influence** on clinical judgment and reasoning—particularly in resource-limited settings—this study **provides preliminary evidence that POCUS may serve as a useful adjunct in hospitalist practice**.”.
  - **Edited:**
    - **Original: “**Similarly, our results exceeded those of Andersen et al. [120], who assessed 199 patients examined by medical residents. They reported changes in the primary diagnosis in 6.5% of cases, while the overall **impact** on clinical management was comparable (35% vs. 39%).”.
    - **Revised to: “**Similarly, our results exceeded those of Andersen et al. [113], who assessed 199 patients examined by medical residents. They reported changes in the primary diagnosis in 6.5% of cases, while the overall **influence** on clinical management was comparable (35% vs. 39%).”
  - **Edited:**
    - **Original: “**Our findings also indicate a greater diagnostic **impact** than Mjolstad et al. [75], who studied the diagnostic effect of cardiac and abdominal screening in 196 hospitalized patients and found a primary diagnosis change in 18.4% of cases.”.
    - **Revised to: “**Our findings also indicate a greater diagnostic **influence** than Mjolstad et al. [75], who studied the diagnostic effect of cardiac and abdominal screening in 196 hospitalized patients and found a primary diagnosis change in 18.4% of cases.”.
  - **Edited:**
    - **Original: “**In our study, even when the primary hypothesis remained unchanged (63% - 66/104), POCUS played **a crucial role** in strengthening diagnostic confidence, with certainty increasing in 36% (24/66) of these cases.”.
    - **Revised to: “**In our study, even when the primary hypothesis remained unchanged (63% - 66/104), POCUS played **an important role** in strengthening diagnostic confidence, with certainty increasing in 36% (24/66) of these cases.”.
  - **Edited:**
    - **Original: “**POCUS also **proved essential** in narrowing differential diagnoses, a particularly valuable function for hospitalists managing complex cases.”
    - **Revised to: “**POCUS also **played** **an essential role** in narrowing differential diagnoses, a particularly valuable function for hospitalists managing complex cases.”
  - **Edited:**
    - **Original: “**These findings highlight how POCUS not only supports diagnostic confirmation but also helps hospitalists efficiently refine differential diagnoses, **ultimately optimizing patient management**.”.
    - **Revised to: “**These findings highlight how POCUS not only supports diagnostic confirmation but also helps hospitalists refine differential diagnoses, **potentially contributing to patient management decisions**.
  - **Edited:**
    - **Original: “**When the primary diagnostic hypothesis changed, POCUS had an even greater **impact**. In 95% (36/38) of these cases, management was modified, **demonstrating** its direct influence on patient care. Additionally, in 84% (32/38) of cases, an alternative diagnosis was ruled out, **reinforcing POCUS’s essential role** in refining differential diagnoses—a key advantage for hospitalists navigating complex decision-making. Confidence levels also shifted **significantly**, with 63% (24/38) of **cases showing a substantial increase** in diagnostic certainty, further emphasizing POCUS’s role in strengthening clinical confidence.”.
    - **Revised to: “**When the primary diagnostic hypothesis changed, POCUS had an even greater **influence**. In 95% (36/38) of these cases, management was modified, **suggesting** its direct influence on patient care. Additionally, in 84% (32/38) of cases, an alternative diagnosis was ruled out, **highlighting POCUS’s role** in refining differential diagnoses—a key advantage for hospitalists navigating complex decision-making. Confidence levels also shifted **notably**, with 63% (24/38) of cases **showing an increase** in diagnostic certainty, further emphasizing POCUS’s role in strengthening clinical confidence.”.
  - **Edited:**
    - **Original: “**This study underscores the **significant impact** of POCUS on the clinical practice of hospitalists, **demonstrating** its **crucial** role in refining clinical reasoning and decision-making. The findings **reveal** that POCUS contributed to a change in the primary diagnostic hypothesis in 37% (38/104) of cases, a modification in clinical management in 39% (41/104), and an increase in diagnostic confidence in 46% (48/104) of cases. Additionally, **its ability to rule out differential diagnoses further enhances the** **precision of** clinical reasoning, which is often complex in hospital medicine. Moreover, POCUS **has even greater** potential to assist hospitalists in settings with limited access to imaging exams, where bedside ultrasound serves as a **valuable tool** for immediate decision-making, **optimizing** patient care and management.”.
    - **Revised to: “Despite these limitations**, our findings underscore the **meaningful influence** of POCUS on the clinical practice of hospitalists, **highlighting** its **important** role in refining clinical reasoning and decision-making. The findings **indicate** that POCUS contributed to a change in the primary diagnostic hypothesis in 37% (38/104) of cases, a modification in clinical management in 39% (41/104), and an increase in diagnostic confidence in 46% (48/104) of cases. Additionally**, the use of POCUS in ruling out differential diagnoses may contribute to refining clinical reasoning,** which is often complex in hospital medicine. Moreover, POCUS **has potential to assist hospitalists** in settings with limited access to imaging exams, where bedside ultrasound serves as a **useful adjunct** to support timely decision-making, **potentially** guiding patient care and management.”.
- **Main text - Conclusion:**
  - **Edited:**
    - **Original: “**This study **revealed** that POCUS had a **meaningful impact** on diagnostic hypotheses, clinical management decisions, and physician confidence. It contributed to clinical reasoning and decision-making in **most** cases, with few instances showing no effect.”.
    - **Revised to: “**This study **suggests** that POCUS **may influence** diagnostic hypotheses, clinical management decisions, and physician confidence. It **appeared** to contribute to clinical reasoning and decision-making in **many** cases, while in **some** instances it showed little or no effect.”.
  - **Edited:**
    - **Original: “**These findings add to the limited body of literature on the application of POCUS in internal medicine, **supporting its utility** as a bedside modality. By **demonstrating** its influence on clinical judgment and reasoning—particularly in resource-limited settings—this study **supports its broader integration into hospitalist practice and highlights the need for standardized training**.”
    - **Revised to: “**These findings add to the limited body of literature on the application of POCUS in internal medicine, **indicating its potential value** as a bedside modality. By **illustrating its possible** influence on clinical judgment and reasoning—particularly in resource-limited settings—this study **provides preliminary evidence that POCUS may serve as a useful adjunct in hospitalist practice**.”.
  - **Edited:**
    - **Original: “**Nevertheless, future research should address **whether enhancements in clinical reasoning facilitated by POCUS** translate into measurable improvements in patient outcomes and healthcare efficiency, such as reduced hospital length of stay, readmission rates, or mortality.”.
    - **Revised to: “Future research should employ stronger designs, such as randomized controlled trials or controlled before-and-after studies with validated outcome measures and objective performance data, to** determine whether enhancements in clinical reasoning facilitated by POCUS translate into measurable improvements in patient outcomes and healthcare efficiency, such as reduced hospital length of stay, readmission rates, or mortality.”.
  - **Edited:**
    - **Original: “**Overall, the results **underscore the relevance of POCUS in supporting** diagnostic accuracy and decision-making in hospital medicine. As clinical demands continue to evolve, incorporating effective bedside approaches remains essential for optimizing patient care.”.
    - **Revised to: “**Overall, **the results should be interpreted as exploratory, emphasizing feasibility and potential utility rather than efficacy**. As clinical demands continue to evolve, incorporating effective bedside approaches like POCUS could ultimately play a supportive role in improving patient care.”.

**Response to Reviewer #4**

**Comment 1:**
*“The chosen study design, however, requires clarification and more precise description. The manuscript currently refers to the study in broad terms, but given the structure — participants undergoing baseline assessment, receiving an educational intervention (POCUS training), and then being reassessed with both OSVE and questionnaires — this aligns most accurately with a prospective quasi-experimental before-and-after design. This classification is important because the study does involve an intervention and measures outcomes before and after its implementation, but it does not include randomization or a parallel control group. Calling it a ‘descriptive prospective study’ would be misleading, as descriptive studies do not assess causal effects or intervention impact. Properly labeling the design as quasi-experimental strengthens methodological transparency and allows readers to appropriately interpret the results.”*

We thank the reviewer for the careful assessment. In response, we removed all pre/post analyses related to the educational session, including any Objective Structured Video Examinations (OSVE) based comparisons, to keep the study focused on our primary objective. The educational session is now presented exclusively as introductory orientation (not an analyzed intervention). Importantly, the OSVE is described only as exposure to standardized clinical vignettes containing Point-of-care Ultrassound (POCUS) images to align participants’ familiarity with image-based content; no OSVE scores or pre/post contrasts enter our analyses. With these changes, the manuscript no longer evaluates intervention effects and does not estimate causal impact. The study therefore fits a prospective descriptive observational design. We also added clarifying language to prevent any implication of a quasi-experimental before-and-after framework.

**Edits implemented:**

- **Abstract – Materials and Methods:**
  - **Edited:**
    - **Original:** “This descriptive prospective study enrolled 19 hospitalists who underwent a brief POCUS training session. Participants completed objective structured video examinations (OSVE) before and after training, and used POCUS in their clinical practice, documenting each application with a standardized form detailing diagnostic hypotheses, management, and confidence levels. Changes in these parameters following POCUS use in real patients were analyzed.”.
    - **Revised to:** “Prospective descriptive study in a transitional care unit enrolling 19 hospitalists. During routine care, clinicians used POCUS at their discretion and, immediately afterward, completed a structured form capturing the clinical question, pre- and post-POCUS diagnostic hypotheses, confidence levels, and management decisions. Per-encounter changes in these parameters were summarized descriptively.”.
- **Abstract - Results:**
  - **Removed:** “While a short training session did not significantly improve OSVE scores, participants reported high satisfaction with the training and perceived utility of POCUS.”.
- **Main text – Material and Methods (Participants and procedures):**
  - **Removed:** “The principal investigator then conducted an initial objective video assessment (OSVE) with 20 questions (maximum score of 10 points) to evaluate participants’ ultrasound interpretation skills (Fig 1).”.
  - **Edited:**
    - **Original:** “Fig 1: Study procedures flowchart. Hospitalists who agreed to participate signed an informed consent form (ICF) and completed a personal data form. **Participants underwent initial objective video assessments (OSVE) and received point-of-care ultrasound (POCUS) training. Following this, they completed a second OSVE** **and a questionnaire assessing their personal impressions and satisfaction.** Each time POCUS was utilized in clinical practice, a detailed record was made using a standardized form.”.
    - **Revised to:** “Fig 1: Study procedures flowchart**.** Hospitalists who agreed to participate signed an informed consent form (ICF) and completed a personal data form. **They then attended a brief POCUS orientation.** During routine care, POCUS was used at clinicians’ discretion. Immediately after each use, participants completed a structured encounter form documenting the clinical question, pre- and post-POCUS diagnostic hypotheses, confidence levels, and management decisions. **At the end of the study, participants completed a perceptions/satisfaction survey.**”.
  - **Edited:**
    - **Original:** “Following the initial assessment, all participants underwent **a 120-minute theoretical and practical POCUS training sessio**n (Fig 1). Topics covered included POCUS applications in clinical practice, equipment use and maintenance, transducer types, image optimization, and interpretation of images from lungs, pleura, heart, abdomen, and vessels. **After training sessions, participants completed a second OSVE to re-evaluate their ultrasound interpretation skills** (Fig 1).”.
    - **Revised to:** “Following the initial assessment, all participants underwent **an introductory educational session on POCUS** (Fig 1), which covered basic applications in clinical practice, equipment use and maintenance, transducer types, image optimization, and interpretation of images from lungs, pleura, heart, abdomen, and vessels. **This session was not based on a standardized protocol; rather, it provided a broad overview of commonly relevant organ systems to internal medicine hospitalists.** **The emphasis was on introducing participants to the potential clinical applications of POCUS rather than on training them to follow a fixed, structured protocol.**”.
- **Main text – Materials and Methods (Statistical analysis):**
  - **Edited:**
    - **Original:** “**The Mann-Whitney Rank Sum test was utilized to examine participants' performance on the OSVE before and after POCUS training, as well as to analyze the “time spent per number of modalities” reported in the POCUS clinical impact forms, in relation to prior hands-on experience and formal POCUS training. The Kruskal-Wallis One-Way Analysis of Variance on Ranks was applied to compare participants’ confidence in their primary diagnostic hypothesis in the first and last forms. Independent samples t-tests were conducted to compare participants' pre-test and post-test OSVE performance according to prior formal POCUS training**. Spearman rank correlation was performed to **evaluate relationships between the remaining variables**.”.
    - **Revised to:** “Spearman rank correlation was performed to **explore associations between continuous and ordinal variables**.”.
- **Main text – Results (Pre and post-tests results):**
  - **Removed:** “For the pre- and post-training test forms (maximum score: 10), 63% (12/19) completed the pre-test, with a median score of 7.25 [5.75; 8.25]. Fifty-three per cent (10/19) completed the post-test, with a median score of 6.25 [5.0; 9.0]. The Mann-Whitney Rank Sum test showed no significant difference between pre-test and post-test performance (T = 105.00; p = 0.970). No significant correlations were found between pre-test scores and participant’s age (R = -0.163; p = 0.603), time since completion of Internal Medicine residency (R = 0.106; p = 0.733), or formal POCUS training (R = -0.155; p = 0.619). No significant correlations were found between post-test scores and participant’s age (R = 0.0864; p = 0.785), time since completion of Internal Medicine residency (R = 0.0308; p = 0.919), or formal POCUS training (R = 0.107; p = 0.759). To assess the relationship between participants’ performance on the pre- and post-test OSVE and prior POCUS training, participants were divided into two groups: those with prior POCUS training and those without. No difference was observed when comparing pre-test scores (T = 0.643; p = 0.535), post-test scores (T = -0.0570; p = 0.956), and the change between pre and post-test scores (T = 0.522; p = 0.616) among both groups. To evaluate whether prior hands-on POCUS experience—measured by the number of times participants reported having performed POCUS before the study—was associated with the change in OSVE scores, we applied the Spearman rank-order correlation. No significant relationship was found between the number of prior POCUS procedures and score variation (R < 0.001; p = 1.000).”.
- **Main text - Discussion:**
  - **Edited:**
    - **Original: “**They had limited formal POCUS training, and their practical experience varied, **leading to differences in skill and confidence**. **While some demonstrated proficiency, many struggled with competence and self-assurance.”.**
    - **Revised to: “**They had limited formal POCUS training, and their practical experience varied, **leading to differences in confidence**.**”.**
  - **Removed:** “No significant difference was found between pre-test and post-test performance (T = 105.00; p = 0.970), indicating that our single short-duration POCUS training did not improve participants' performance in image interpretation on the OSVE. This suggests that the skills required for POCUS image interpretation likely demand more extensive training and hands-on practice. Additionally, several factors may have contributed to this finding. First, our study was not specifically designed as a medical education study, which may have impacted on the effectiveness of the training approach. Second, the assessment tool we used may have been too challenging, as reflected in the participants' test scores, potentially limiting the ability to detect improvements. Additionally, the limited participation rates in the pre- and post-training OSVE —63% and 53% respectively—may have diluted the observed impact of the training on participants' knowledge. Finally, the alignment between the assessment tool and the content covered in the training may not have been optimal, which could have influenced the measured outcomes. This study found no statistically significant differences in OSVE performance between participants with and without prior formal POCUS training, either in the pre-test, post-test, or in the variation between them. Likewise, no correlation was observed between the number of prior POCUS procedures performed and changes in test scores. These findings suggest that previous exposure to POCUS—whether formal or practical—was not associated with better performance in this structured assessment. A possible explanation is that the OSVE may evaluate broader skills such as clinical reasoning and integration of ultrasound findings into decision-making, which may not be fully developed through prior training alone. Additionally, variability in the type and depth of previous training, as well as the small sample size, may have limited the detection of differences. While prior formal POCUS training was not associated with improvements in time efficiency—measured by the change in time-per-modality ratio between each participant’s first and last form—greater hands-on experience, defined as the self-reported number of times participants had performed POCUS prior to the study, showed a significant negative correlation with this variable. This means that participants with more prior POCUS experience not only started out faster (lower time-per-modality ratios in their first forms) but also showed greater improvement in efficiency over time. These findings suggest that repeated practical exposure may play a more important role in enhancing efficiency during ultrasound use than formal training alone.”.
  - **Edited:**
    - **Original:** “Despite the lack of statistical improvement in confidence, the survey results revealed a highly positive perception of **POCUS training**.”.
    - **Revised to:** “The survey results revealed a highly positive perception of **brief POCUS orientation session**.”.

**Comment 2:**
*“I have concerns regarding the measurement of two key process variables: (a) time spent performing the POCUS exam, and (b) quality of images obtained. According to the data collection form, both variables were captured through participant self-report rather than objective measurement. For time spent, it is unclear whether this was based on a stopwatch or structured observation; as presented, the data seem to rely entirely on participants’ subjective estimation. Similarly, the assessment of image quality was based on participants’ own descriptions without saving or independently reviewing the images. This introduces a high risk of recall bias and self-report bias. Moreover, not storing images for subsequent blinded quality review represents a missed opportunity to validate this outcome objectively. These limitations should be explicitly acknowledged in the manuscript, as they constrain the strength of the conclusions regarding feasibility and quality of POCUS implementation.”*

We thank the reviewer for these important observations. In the revised manuscript, we clarified in the Methods that both process variables were self-reported rather than objectively measured. Specifically, we now state that exam duration was reported as an estimated time by the participant, not measured with a stopwatch or structured observation. Similarly, we emphasize that image quality was based solely on participants’ self-reported perceptions. In addition, we made it explicit that POCUS images were not recorded, which precluded independent verification of acquisition quality, diagnostic accuracy, or related management decisions.

We also revised the Discussion to explicitly acknowledge these issues as limitations. We highlight that reliance on participants’ estimations introduces a risk of recall bias, while self-reported image quality without external review introduces self-report bias. Furthermore, we note that not storing images prevented objective validation of findings, interobserver reliability assessment, or concordance with gold-standard criteria. These aspects are now clearly recognized as factors that constrain the strength of our conclusions regarding feasibility and quality of POCUS implementation.

**Edits implemented:**

- **Materials and Methods (Participants and procedures):**
  - **Edited:**
    - **Original:** “After each use, participants completed a detailed form (Fig 1) documenting the reason for using POCUS, the initial diagnostic hypothesis, planned actions, the ultrasound modalities used—chosen from seven options (cardiac, pulmonary, abdominal, inferior vena cava, femoral and popliteal veins, bladder, and other), with the possibility of selecting multiple modalities per form—, **exam duration,** findings, final diagnostic hypothesis, and subsequent clinical decisions (S2 appendix B). **Since POCUS images were not recorded, it was not possible to independent verify the accuracy of the diagnostic findings or the clinical management decisions informed by them**.”.
    - **Revised to**: “**Immediately** after each use, participants completed a detailed form (Fig 1) documenting the reason for using POCUS, the initial diagnostic hypothesis, planned actions, the ultrasound modalities used—chosen from seven options (cardiac, pulmonary, abdominal, inferior vena cava, femoral and popliteal veins, bladder, and other), with the possibility of selecting multiple modalities per form—, **the estimated duration of the exam (self-reported), the perceived quality of the images obtained (self-reported)**, findings, final diagnostic hypothesis, and subsequent clinical decisions (S2 appendix B). …**In addition, because POCUS images were not recorded, independent verification of diagnostic accuracy, image quality, and related clinical decisions was not feasible.**”
- **Discussion:**
  - **Edited:**
    - **Original:** “The limitations of this study include the lack of assessment of the impact of POCUS on robust clinical outcomes, such as length of stay, readmission rates, and mortality. Another limitation is the sample size, which may affect the generalizability of the results and increase susceptibility to bias. **Additionally, the POCUS images were not recorded, which precluded independent verification of image acquisition quality and the accuracy of the corresponding diagnostic findings or management decisions. Consequently, we were unable to assess interobserver reliability or confirm whether image interpretation was consistent with accepted diagnostic criteria.** Finally, the absence of gold-standard complementary exams to confirm POCUS findings represents an important limitation, as interpretation can be influenced by operator experience and patient conditions.”
    - **Revised to:** “This study has some limitations. First, we did not assess the impact of POCUS on robust clinical outcomes such as length of stay, readmission rates, or mortality. Second, the small sample size may limit the generalizability of the results and increase susceptibility to bias. **Third, POCUS images were not recorded, precluding independent review of acquisition and interpretation, and preventing evaluation of diagnostic accuracy, interobserver reliability, or concordance with gold-standard criteria. Decisions were therefore based solely on participants’ self-reported findings, which may have led to overestimation of perceived safety and influence. In addition, process variables such as exam duration and image quality were also self-reported rather than objectively measured. Exam time was estimated by participants, raising the risk of recall bias, while image quality was judged subjectively without external validation, introducing self-report bias.** Another limitation relates to the data collection form, which was developed by the authors based on previous studies and study objectives but was not formally validated; as such, the accuracy and interpretability of the captured constructs may be constrained. Finally, open-ended responses were categorized by a single investigator without independent coding or inter-rater reliability assessment, which may introduce subjective bias. **These aspects should be considered when interpreting the feasibility and quality of POCUS implementation in our study.**”.

**Comment 3:**
*“In addition, the manuscript does not provide sufficient information on the development and validation of the data collection forms. It is unclear whether the instruments underwent a systematic process of construction (e.g., based on literature review, expert consultation) and whether any procedures to establish evidence of content validity were performed. Without such information, it is difficult to assess whether the forms adequately captured the intended constructs (e.g., diagnostic hypotheses, management changes, confidence levels, time spent, and image quality). The absence of reported validity evidence represents an important limitation, as the accuracy and interpretability of the findings rely heavily on the adequacy of these instruments.”*

We thank the reviewer for this valuable observation. In the revised manuscript, we clarified in the Methods that the data collection form was collaboratively developed by the authors, informed by prior studies in internal medicine/hospital medicine POCUS, and tailored to the specific objectives of this study. We also make explicit that the instrument was not formally validated and that no psychometric procedures (e.g., content validity assessment, inter-rater reliability testing) were performed.

Furthermore, we revised the Discussion to explicitly acknowledge this limitation. We highlight that, because the form was author-developed and not validated, the accuracy and interpretability of the constructs captured (diagnostic hypotheses, management changes, confidence levels, exam duration, and image quality) may be constrained. This limitation is now clearly stated alongside other methodological caveats.

**Edits implemented:**

- **Materials and Methods (Participants and procedures):**
  - **Added:** “The form was collaboratively developed by the authors, based on previous studies evaluating the diagnostic and clinical influence of POCUS in internal medicine and hospitalist settings, and adapted to the specific objectives of this study. However, the instrument was not formally validated, and no systematic psychometric evaluation was performed.”
- **Discussion:**
  - **Edited:**
    - **Original:** “The limitations of this study include the lack of assessment of the impact of POCUS on robust clinical outcomes, such as length of stay, readmission rates, and mortality. Another limitation is the sample size, which may affect the generalizability of the results and increase susceptibility to bias. Additionally, the POCUS images were not recorded, which precluded independent verification of image acquisition quality and the accuracy of the corresponding diagnostic findings or management decisions. Consequently, we were unable to assess interobserver reliability or confirm whether image interpretation was consistent with accepted diagnostic criteria. Finally, the absence of gold-standard complementary exams to confirm POCUS findings represents an important limitation, as interpretation can be influenced by operator experience and patient conditions.”
    - **Revised to:** “This study has some limitations. First, we did not assess the impact of POCUS on robust clinical outcomes such as length of stay, readmission rates, or mortality. Second, the small sample size may limit the generalizability of the results and increase susceptibility to bias. Third, POCUS images were not recorded, precluding independent review of acquisition and interpretation, and preventing evaluation of diagnostic accuracy, interobserver reliability, or concordance with gold-standard criteria. Decisions were therefore based solely on participants’ self-reported findings, which may have led to overestimation of perceived safety and influence. In addition, process variables such as exam duration and image quality were also self-reported rather than objectively measured. Exam time was estimated by participants, raising the risk of recall bias, while image quality was judged subjectively without external validation, introducing self-report bias. **Another limitation relates to the data collection form, which was developed by the authors based on previous studies and study objectives but was not formally validated; as such, the accuracy and interpretability of the captured constructs may be constrained.** Finally, open-ended responses were categorized by a single investigator without independent coding or inter-rater reliability assessment, which may introduce subjective bias. **These aspects should be considered when interpreting the feasibility and quality of POCUS implementation in our study.**”.

**Comment 4:**
*“In addition, it is unclear whether the POCUS training and subsequent examinations followed a standardized protocol, such as the RUSH protocol. The data collection form included the following examination categories: cardiac, pulmonary, abdominal, inferior vena cava, femoral and popliteal veins, bladder, and ‘other’. The RUSH protocol encompasses cardiac function and contractility assessment, evaluation of intravascular volume status via the inferior vena cava, identification of free fluid in the abdomen, lung assessment for pneumothorax or pulmonary embolism, and lower limb DVT screening. Given this overlap, it should be explicitly stated whether the investigators adopted the RUSH protocol in its entirety, used a modified version, or employed another structured approach. If the RUSH protocol was not used, the authors should clearly describe which anatomical regions and clinical targets were prioritized in both training and clinical application."*

We thank the reviewer for raising this point. In the revised Methods, we clarified that neither the educational session nor the subsequent clinical examinations followed a standardized protocol such as RUSH. Examinations were performed entirely at the discretion of each physician, guided by the specific clinical question in each encounter.

The educational session provided a broad, introductory overview of POCUS for hospitalists, covering lungs/pleura, heart, abdomen, inferior vena cava, femoral/popliteal veins, and bladder, as well as basic equipment handling and image optimization. This was intended as an orientation, not as training in a fixed algorithm. The overlap between our data collection categories and components of the RUSH protocol reflects the fact that these organ systems are commonly relevant in hospital medicine POCUS, rather than adoption of RUSH or any other structured pathway.

**Edits implemented:**

- **Materials and Methods (Participants and Procedures):**
  - **Edited:**
    - **Original: “**Following the initial assessment, all participants underwent a 120-minute theoretical and practical POCUS training session (Fig 1). Topics covered included POCUS applications in clinical practice, equipment use and maintenance, transducer types, image optimization, and interpretation of images from lungs, pleura, heart, abdomen, and vessels.”.
    - **Revised to “**Following the initial assessment, all participants underwent an introductory educational session on POCUS (Fig 1), which covered basic applications in clinical practice, equipment use and maintenance, transducer types, image optimization, and interpretation of images from lungs, pleura, heart, abdomen, and vessels. **This session was not based on a standardized protocol; rather, it provided a broad overview of commonly relevant organ systems to internal medicine hospitalists. The emphasis was on introducing participants to the potential clinical applications of POCUS rather than on training them to follow a fixed, structured protocol**.”.
  - **Edited:**
    - **Original: “**Throughout the study, participants had access to a portable ultrasound machine (Mindray DC-80A/2022) and used it as needed in clinical practice.”
    - **Revised to:** “Throughout the study, participants had access to a portable ultrasound machine (Mindray DC-80A/2022) and used it as needed in clinical practice. **Examinations were performed at the discretion of each physician according to the clinical question, without following a predefined or standardized protocol**.”

**Comment 5:**
*“The manuscript states that responses to open-ended questions were kept in their original format, but it does not describe the procedures adopted for their analysis. It remains unclear whether these responses were analyzed qualitatively (e.g., through thematic or content analysis), coded systematically by independent reviewers, or simply presented as anecdotal remarks. Without a clear description of the analytic approach, the contribution of these data to the study’s findings is difficult to assess. The authors should specify the method used to analyze the open-ended responses, including whether any coding framework, inter-rater reliability, or software tools were applied. If the responses were not systematically analyzed, this should be acknowledged as a limitation.”*

We thank the reviewer for this important observation. In the revised manuscript, we clarified in the Methods that open-ended responses and relevant closed-ended items were transcribed into a structured Excel sheet and coded by the principal investigator for each form. Binary variables were created to capture changes in diagnostic hypotheses, diagnostic confidence, addition or exclusion of alternative diagnoses, incidental findings, and management changes. For diagnostic confidence, responses were recoded into three categories (Likert 1–2, 3, and 4–5) to define shifts across confidence levels. These derived variables were analyzed descriptively as categorical data. Open-text responses were also reviewed to extract brief contextual information; however, no formal qualitative analysis was performed, and no independent coding, inter-rater reliability procedures, or software-assisted thematic analysis were applied.

We also revised the Discussion to explicitly acknowledge this as a limitation. Specifically, we note that open-ended responses were categorized by a single investigator without independent review, coding framework validation, or reliability assessment, which may have introduced subjective bias. This caveat is now clearly presented alongside the other methodological limitations to aid readers in interpreting the findings.

**Edits implemented:**

- **Materials and Methods (Statistical analysis):**
  - **Removed:** “Open-ended question responses were kept in their original format.”
  - **Added:** “In addition, open-ended question responses and relevant closed-ended items from the POCUS clinical-use form (S2 Appendix B) were transcribed into a structured Excel sheet and coded by the principal investigator for each form. Open-ended items used in the analyses were: primary diagnostic hypothesis (pre/post-POCUS), differential diagnoses (pre/post-POCUS), planned management (pre/post-POCUS), expected findings, and POCUS findings. The closed-ended item used was the diagnostic confidence in the primary hypothesis (pre/post-POCUS). From these fields, we created binary indicators (yes/no) for: change in the primary diagnostic hypothesis; change in diagnostic confidence; addition of an alternative diagnosis; exclusion of an alternative diagnosis; incidental findings; and change in management. Change in diagnostic confidence was defined as a shift between the three recoded Likert categories (value 1 = Likert 1–2; value 2 = Likert 3; value 3 = Likert 4–5). These derived variables were summarized descriptively as categorical data, and no formal qualitative analysis was performed. Open-text responses were also reviewed to extract brief contextual information.”.
- **Discussion:**
  - **Edited:**
    - **Original:** “The limitations of this study include the lack of assessment of the impact of POCUS on robust clinical outcomes, such as length of stay, readmission rates, and mortality. Another limitation is the sample size, which may affect the generalizability of the results and increase susceptibility to bias. Additionally, the POCUS images were not recorded, which precluded independent verification of image acquisition quality and the accuracy of the corresponding diagnostic findings or management decisions. Consequently, we were unable to assess interobserver reliability or confirm whether image interpretation was consistent with accepted diagnostic criteria. Finally, the absence of gold-standard complementary exams to confirm POCUS findings represents an important limitation, as interpretation can be influenced by operator experience and patient conditions.”
    - **Revised to:** “This study has some limitations. First, we did not assess the impact of POCUS on robust clinical outcomes such as length of stay, readmission rates, or mortality. Second, the small sample size may limit the generalizability of the results and increase susceptibility to bias. Third, POCUS images were not recorded, precluding independent review of acquisition and interpretation, and preventing evaluation of diagnostic accuracy, interobserver reliability, or concordance with gold-standard criteria. Decisions were therefore based solely on participants’ self-reported findings, which may have led to overestimation of perceived safety and influence. In addition, process variables such as exam duration and image quality were also self-reported rather than objectively measured. Exam time was estimated by participants, raising the risk of recall bias, while image quality was judged subjectively without external validation, introducing self-report bias. Another limitation relates to the data collection form, which was developed by the authors based on previous studies and study objectives but was not formally validated; as such, the accuracy and interpretability of the captured constructs may be constrained. **Finally, open-ended responses were categorized by a single investigator without independent coding or inter-rater reliability assessment, which may introduce subjective bias.** **These aspects should be considered when interpreting the feasibility and quality of POCUS implementation in our study.**”.

**Comment 6:**
*“The sample size rationale estimates a single post-POCUS proportion with 95% confidence and 10% margin of error, assuming p=0.20, yielding ≈62 forms. This is acceptable for estimating a single proportion. However, because multiple forms were submitted by the same hospitalists, observations are clustered. The calculation should therefore include a design-effect inflation based on the average forms per clinician and an assumed ICC. Using 5.5 forms/physician and plausible ICCs (0.05–0.10) increases the requirement to approximately 76–90 forms. The 104 forms collected likely satisfy this, but the manuscript should explicitly report the clustering adjustment (DEFF and ICC). In addition, the finite-population correction used should reflect the finite number of eligible encounters in the unit rather than the number of internists in the state; otherwise, FPC should be omitted.”*

We thank the reviewer for this important observation. We agree that multiple forms submitted by the same physician introduce clustering, requiring adjustment. In the revised manuscript, we now explicitly report this. Because it is not possible to determine the finite number of eligible encounters within the Transitional Care Unit, we omitted the finite-population correction, as recommended by the reviewer, and applied the single-proportion formula assuming an infinite population.

In addition, we incorporated the reviewer’s recommendation to include a design-effect adjustment based on the average number of forms per physician and an assumed intraclass correlation coefficient (ICC). Since there are no prior studies to inform a precise ICC value in this context, we opted for a conservative estimate of ICC = 0.10. Using the observed average of 5.5 forms per hospitalist in our dataset, this adjustment increased the required sample size from 62 to approximately 90 forms. Our final dataset of 104 forms exceeded this threshold, thus satisfying the adjusted requirement.

**Edits implemented:**

- **Materials and Methods (Sample size):**
  - **Edited:**
    - **Original:** “The sample size was calculated using the formula for proportions with finite population correction, appropriate for binary nominal outcomes[115–118]. The primary outcome was defined as a change in the main diagnostic hypothesis and/or management plan. These outcome variables are categorical with two mutually exclusive categories, corresponding to the presence or absence of change. Assumptions included a 95% confidence level, 10% margin of error, and a finite population of 15,860 internal medicine physicians in the state of São Paulo[119]. An expected proportion of 20% was used, based on prior studies[64,72,75,108,120] indicating that POCUS results in a change in diagnostic reasoning or management in approximately 20% of cases. The calculated required sample size was 61 forms on POCUS clinical impact (S2 appendix B).
    - **Revised to:** “The sample size was calculated using the single-proportion formula for binary outcomes. The primary outcome was defined as a change in the main diagnostic hypothesis and/or management plan—both coded as mutually exclusive categories (change vs. no change). Assumptions included a 95% confidence level, a 10 percentage-point margin of error, and an expected proportion of 20% based on prior studies [64,72,75,108,120], yielding a minimum of 62 forms (S2 Appendix B). **Because multiple forms were submitted per physician, observations were treated as clustered. Accordingly, we applied a design-effect adjustment using an average cluster size of 5.5 forms per hospitalist (as observed in our data, presented below) and a conservative intraclass correlation coefficient of 0.10, resulting in an adjusted requirement of approximately 90 forms (S2 Appendix B)**.”.

**Comment 7:**
*“The study demonstrates positive trends but does so with methodological fragility. The results should be framed more cautiously, emphasizing feasibility and potential utility rather than efficacy. Stronger study designs (e.g., randomized controlled trials or controlled before-and-after studies with validated outcome measures and objective performance data) would be necessary to substantiate these findings”*

We fully agree and have revised the **entire manuscript** to adopt a more cautious framing. The revised text emphasizes feasibility and perceived influence on clinical reasoning. We now state that stronger study designs with validated protocols and competency assessment are required to confirm these findings.

**Edits implemented:**

- **Article title:**
  - **Edited:**
    - **Original:** “Assessing the clinical relevance of point-of-care ultrasound for hospitalists: **impact** on clinical reasoning and decision-making”.
    - **Revised to:** “Assessing the clinical relevance of point-of-care ultrasound for hospitalists: **influence** on clinical reasoning and decision-making”.
- **Abstract - Introduction:**
  - **Edited:**
    - **Original: “**Point-of-care ultrasonography (POCUS) is increasingly recognized in internal medicine, yet its **impact** on hospitalists' clinical reasoning remains underexplored.”
    - **Revised to: “**Point-of-care ultrasonography (POCUS) is increasingly recognized in internal medicine, yet its **influence** on hospitalists' clinical reasoning remains underexplored.”
- **Abstract - Results:**
  - **Edited:**
    - **Original:** “Notably, POCUS had no discernible **impact** in only 13% (14/104) of cases.”.
    - **Revised to:** “Notably, POCUS had no discernible **influence** in only 13% (14/104) of cases.”.
- **Abstract - Conclusion:**
  - **Edited:**
    - **Original:** “This study **demonstrates** that POCUS **significantly influences** clinical reasoning and decision-making among hospitalists in a setting with limited imaging resources.”.
    - **Revised to: “**This study **suggests** that POCUS **may influence** clinical reasoning and decision-making among hospitalists in a setting with limited imaging resources.”
  - **Edited:**
    - **Original:** “These findings **highlight** the valuable role of POCUS in enhancing bedside **diagnostics** and patient management in internal medicine, underscoring the need for its integration into training programs and clinical practice.”.
    - **Revised to:** “These findings **suggest** that POCUS **may play a supportive** role in bedside **assessment** and patient management in internal medicine, underscoring the need for further research and structured training **before broader integration**.”.
- **Main text - Introduction:**
  - **Edited:**
    - **Original: “**Although studies suggest that POCUS leads to unexpected diagnoses and management changes among hospitalists, its direct **impact** on clinical reasoning remains understudied.”.
    - **Revised to: “**Although studies suggest that POCUS leads to unexpected diagnoses and management changes among hospitalists, its direct **influence** on clinical reasoning remains understudied.”.
- **Main text subtitles - Results:**
  - **Edited:**
    - **Original: “Impact** of POCUS on clinical reasoning and decision-making”
    - **Revised to: “Influence** of POCUS on clinical reasoning and decision-making”
- **Main text – Results (Clinical application of POCUS):**
  - **Edited:**
    - **Original: “**In total, 104 forms on POCUS clinical **impact** were completed by 19 participants throughout the study…”.
    - **Revised to: “**In total, 104 forms on POCUS clinical **use** were completed by 19 participants throughout the study…”.
- **Main text – Results (Influence of POCUS on clinical reasoning and decision making):**
  - **Edited:**
    - **Original: “**Of the 104 completed forms, only 14/104 (13%) showed no **impact** of POCUS on clinical reasoning or decision-making.”.
    - **Revised to: “**Of the 104 completed forms, only 14/104 (13%) showed no **influence** of POCUS on clinical reasoning or decision-making.”.
  - **Edited:**
    - **Original:** “Fig. 4: Diagnostic **impact**”.
    - **Revised to**: “Fig. 4: Diagnostic **influence**”.
  - **Edited:**
    - **Original: “**The remaining 24/25 (96%) recorded **significant** increases…”.
    - **Revised to: “**The remaining 24/25 (96%) **recorded** increases…”.
- **Main text - Discussion:**
  - **Edited:**
    - **Original: “**Our study **demonstrates** that POCUS **has a significant impact** on clinical reasoning and decision-making.”
    - **Revised to: “**Our study **suggests** that POCUS **may** influence clinical reasoning and decision-making.”
  - **Edited:**
    - **Original: “**Compared to Cid-Serra et al. [72], who conducted a systematic review encompassing six studies with a total of 1,836 patients, our study found a higher rate of primary diagnostic hypothesis changes (37% vs. 18%), though clinical management **impact** was similar (39% vs. 37–52.1%).”
    - **Revised to:** “Compared to Cid-Serra et al. [72], who conducted a systematic review encompassing six studies with a total of 1,836 patients, our study found a higher rate of primary diagnostic hypothesis changes (37% vs. 18%), though clinical management **influence** was similar (39% vs. 37–52.1%).”
  - **Edited:**
    - **Original: “**Similarly, our results exceeded those of Andersen et al. [120], who assessed 199 patients examined by medical residents. They reported changes in the primary diagnosis in 6.5% of cases, while the overall **impact** on clinical management was comparable (35% vs. 39%).”
    - **Revised to: “**Similarly, our results exceeded those of Andersen et al. [113], who assessed 199 patients examined by medical residents. They reported changes in the primary diagnosis in 6.5% of cases, while the overall **influence** on clinical management was comparable (35% vs. 39%).”
  - **Edited:**
    - **Original: “**These findings add to the limited body of literature on the application of POCUS in internal medicine, **supporting** its utility as a bedside modality. By demonstrating **its influence** on clinical judgment and reasoning—particularly in resource-limited settings—this study **supports its broader integration into hospitalist practice and highlights the need for standardized training**.”.
    - **Revised to: “**These findings add to the limited body of literature on the application of POCUS in internal medicine, **indicating its potential value** as a bedside modality. By illustrating **its possible influence** on clinical judgment and reasoning—particularly in resource-limited settings—this study **provides preliminary evidence that POCUS may serve as a useful adjunct in hospitalist practice**.”.
  - **Edited:**
    - **Original: “**Similarly, our results exceeded those of Andersen et al. [120], who assessed 199 patients examined by medical residents. They reported changes in the primary diagnosis in 6.5% of cases, while the overall **impact** on clinical management was comparable (35% vs. 39%).”.
    - **Revised to: “**Similarly, our results exceeded those of Andersen et al. [113], who assessed 199 patients examined by medical residents. They reported changes in the primary diagnosis in 6.5% of cases, while the overall **influence** on clinical management was comparable (35% vs. 39%).”
  - **Edited:**
    - **Original: “**Our findings also indicate a greater diagnostic **impact** than Mjolstad et al. [75], who studied the diagnostic effect of cardiac and abdominal screening in 196 hospitalized patients and found a primary diagnosis change in 18.4% of cases.”.
    - **Revised to: “**Our findings also indicate a greater diagnostic **influence** than Mjolstad et al. [75], who studied the diagnostic effect of cardiac and abdominal screening in 196 hospitalized patients and found a primary diagnosis change in 18.4% of cases.”.
  - **Edited:**
    - **Original: “**In our study, even when the primary hypothesis remained unchanged (63% - 66/104), POCUS played **a crucial role** in strengthening diagnostic confidence, with certainty increasing in 36% (24/66) of these cases.”.
    - **Revised to: “**In our study, even when the primary hypothesis remained unchanged (63% - 66/104), POCUS played **an important role** in strengthening diagnostic confidence, with certainty increasing in 36% (24/66) of these cases.”.
  - **Edited:**
    - **Original: “**POCUS also **proved essential** in narrowing differential diagnoses, a particularly valuable function for hospitalists managing complex cases.”
    - **Revised to: “**POCUS also **played** **an essential role** in narrowing differential diagnoses, a particularly valuable function for hospitalists managing complex cases.”
  - **Edited:**
    - **Original: “**These findings highlight how POCUS not only supports diagnostic confirmation but also helps hospitalists efficiently refine differential diagnoses, **ultimately optimizing patient management**.”.
    - **Revised to: “**These findings highlight how POCUS not only supports diagnostic confirmation but also helps hospitalists refine differential diagnoses, **potentially contributing to patient management decisions**.
  - **Edited:**
    - **Original: “**When the primary diagnostic hypothesis changed, POCUS had an even greater **impact**. In 95% (36/38) of these cases, management was modified, **demonstrating** its direct influence on patient care. Additionally, in 84% (32/38) of cases, an alternative diagnosis was ruled out, **reinforcing POCUS’s essential role** in refining differential diagnoses—a key advantage for hospitalists navigating complex decision-making. Confidence levels also shifted **significantly**, with 63% (24/38) of **cases showing a substantial increase** in diagnostic certainty, further emphasizing POCUS’s role in strengthening clinical confidence.”.
    - **Revised to: “**When the primary diagnostic hypothesis changed, POCUS had an even greater **influence**. In 95% (36/38) of these cases, management was modified, **suggesting** its direct influence on patient care. Additionally, in 84% (32/38) of cases, an alternative diagnosis was ruled out, **highlighting POCUS’s role** in refining differential diagnoses—a key advantage for hospitalists navigating complex decision-making. Confidence levels also shifted **notably**, with 63% (24/38) of cases **showing an increase** in diagnostic certainty, further emphasizing POCUS’s role in strengthening clinical confidence.”.
  - **Edited:**
    - **Original: “**This study underscores the **significant impact** of POCUS on the clinical practice of hospitalists, **demonstrating** its **crucial** role in refining clinical reasoning and decision-making. The findings **reveal** that POCUS contributed to a change in the primary diagnostic hypothesis in 37% (38/104) of cases, a modification in clinical management in 39% (41/104), and an increase in diagnostic confidence in 46% (48/104) of cases. Additionally, **its ability to rule out differential diagnoses further enhances the** **precision of** clinical reasoning, which is often complex in hospital medicine. Moreover, POCUS **has even greater** potential to assist hospitalists in settings with limited access to imaging exams, where bedside ultrasound serves as a **valuable tool** for immediate decision-making, **optimizing** patient care and management.”.
    - **Revised to: “Despite these limitations**, our findings underscore the **meaningful influence** of POCUS on the clinical practice of hospitalists, **highlighting** its **important** role in refining clinical reasoning and decision-making. The findings **indicate** that POCUS contributed to a change in the primary diagnostic hypothesis in 37% (38/104) of cases, a modification in clinical management in 39% (41/104), and an increase in diagnostic confidence in 46% (48/104) of cases. Additionally**, the use of POCUS in ruling out differential diagnoses may contribute to refining clinical reasoning,** which is often complex in hospital medicine. Moreover, POCUS **has potential to assist hospitalists** in settings with limited access to imaging exams, where bedside ultrasound serves as a **useful adjunct** to support timely decision-making, **potentially** guiding patient care and management.”.
- **Main text - Conclusion:**
  - **Edited:**
    - **Original: “**This study **revealed** that POCUS had a **meaningful impact** on diagnostic hypotheses, clinical management decisions, and physician confidence. It contributed to clinical reasoning and decision-making in **most** cases, with few instances showing no effect.”.
    - **Revised to: “**This study **suggests** that POCUS **may influence** diagnostic hypotheses, clinical management decisions, and physician confidence. It **appeared** to contribute to clinical reasoning and decision-making in **many** cases, while in **some** instances it showed little or no effect.”.
  - **Edited:**
    - **Original: “**These findings add to the limited body of literature on the application of POCUS in internal medicine, **supporting its utility** as a bedside modality. By **demonstrating** its influence on clinical judgment and reasoning—particularly in resource-limited settings—this study **supports its broader integration into hospitalist practice and highlights the need for standardized training**.”
    - **Revised to: “**These findings add to the limited body of literature on the application of POCUS in internal medicine, **indicating its potential value** as a bedside modality. By **illustrating its possible** influence on clinical judgment and reasoning—particularly in resource-limited settings—this study **provides preliminary evidence that POCUS may serve as a useful adjunct in hospitalist practice**.”.
  - **Edited:**
    - **Original: “**Nevertheless, future research should address **whether enhancements in clinical reasoning facilitated by POCUS** translate into measurable improvements in patient outcomes and healthcare efficiency, such as reduced hospital length of stay, readmission rates, or mortality.”.
    - **Revised to: “Future research should employ stronger designs, such as randomized controlled trials or controlled before-and-after studies with validated outcome measures and objective performance data, to** determine whether enhancements in clinical reasoning facilitated by POCUS translate into measurable improvements in patient outcomes and healthcare efficiency, such as reduced hospital length of stay, readmission rates, or mortality.”.
  - **Edited:**
    - **Original: “**Overall, the results **underscore the relevance of POCUS in supporting** diagnostic accuracy and decision-making in hospital medicine. As clinical demands continue to evolve, incorporating effective bedside approaches remains essential for optimizing patient care.”.
    - **Revised to: “**Overall, **the results should be interpreted as exploratory, emphasizing feasibility and potential utility rather than efficacy**. As clinical demands continue to evolve, incorporating effective bedside approaches like POCUS could ultimately play a supportive role in improving patient care.”.

**Final response to reviewers**

In addition to the revisions made in direct response to the reviewers’ comments, we implemented a few further improvements to enhance clarity and readability.

First, we edited the following sentence to improve the fluency of the text:
*“Upon study completion, participants* ***completed*** *a survey (Fig 1) assessing their satisfaction with POCUS training, its usefulness and relevance in clinical practice, confidence in* ***using the method****, and any* ***challenges encountered with the equipment*** *(S3 Appendix C)”*
to:
*“Upon study completion, participants* ***filled out*** *a survey (Fig 1) assessing their satisfaction with POCUS training, its usefulness and relevance in clinical practice, confidence in* ***performing POCUS****, and any* ***equipment-related challenges*** *(S3 Appendix C).”*

Second, because exam-time data did not follow a normal distribution according to the Shapiro–Wilk test (as described in the Methods), we replaced the mean with the median and interquartile range for the per-patient time:
*“The total time spent using POCUS was 935 minutes, with a median of 10 minutes per patient [5.00; 10.00].”*

Third, to facilitate interpretation, we added *Table 2 — POCUS Influence on Diagnostic Reasoning and Decision-Making When the Primary Diagnostic Hypothesis Was Maintained vs. Changed*, which summarizes diagnostic confidence, ruling out of hypotheses, incidental findings, addition of hypotheses, and management changes, stratified by whether the primary diagnostic hypothesis was maintained or changed.

Finally, we decided to add a new reflection to the Discussion, acknowledging broader implications of our findings for medical education and practice. We therefore included the following paragraph:
*“Beyond the direct findings, our study highlights broader implications for hospital medicine. First, the frequent use of POCUS by hospitalists in diverse scenarios suggests that it is becoming integrated into routine reasoning processes. By influencing diagnostic hypotheses, management strategies, and confidence levels, POCUS appears to shape clinical reasoning in meaningful ways. However, most participants reported limited prior formal training, reflecting the well-documented gap in structured POCUS education within internal medicine residency programs. This gap raises important concerns. The use of POCUS without adequate training may increase the risk of misinterpretation and diagnostic error, undermining the tool’s potential benefits. Previous studies have emphasized that appropriate supervision, competency standards, and validated curricula are essential to ensure safe and effective integration of POCUS into practice [93,108,110–112]. Therefore, our findings reinforce the need for internal medicine societies and residency program directors to systematically consider how POCUS training should be incorporated into education and clinical governance frameworks.”*

**Closing Statement**

We again thank the reviewers for their thoughtful and detailed critiques. We believe the revisions have substantially improved the clarity, methodological transparency, and caution of our manuscript.
